# High-Sensitivity and High-Resolution In Situ Hybridization of Coding and Long Non-coding RNAs in Vertebrate Ovaries and Testes

**DOI:** 10.1186/s12575-018-0071-z

**Published:** 2018-03-01

**Authors:** Natsumi Takei, Takuma Nakamura, Shohei Kawamura, Yuki Takada, Yui Satoh, Atsushi P. Kimura, Tomoya Kotani

**Affiliations:** 1 Biosystems Science Course, Graduate School of Life Science, Hokkaido University, Sapporo, 060-0810 Japan; 2 Department of Biological Sciences, Faculty of Science, Hokkaido University, North 10 West 8, Sapporo, Hokkaido 060-0810 Japan

**Keywords:** RNA localization, Post-transcriptional regulation, Germ cell, Tissue, Organ

## Abstract

**Background:**

Subcellular localization of coding and non-coding RNAs has emerged as major regulatory mechanisms of gene expression in various cell types and many organisms. However, techniques that enable detection of the subcellular distribution of these RNAs with high sensitivity and high resolution remain limited, particularly in vertebrate adult tissues and organs. In this study, we examined the expression and localization of mRNAs encoding Pou5f1/Oct4, Mos, Cyclin B1 and Deleted in Azoospermia-like (Dazl) in zebrafish and mouse ovaries by combining tyramide signal amplification (TSA)-based in situ hybridization with paraffin sections which can preserve cell morphology of tissues and organs at subcellular levels. In addition, the distribution of a long non-coding RNA (lncRNA), *lncRNA-HSVIII*, in mouse testes was examined by the same method.

**Results:**

The mRNAs encoding Mos, Cyclin B1 and Dazl were found to assemble into distinct granules that were distributed in different subcellular regions of zebrafish and mouse oocytes, suggesting conserved and specific regulations of these mRNAs. The *lncRNA-HSVIII* was first detected in the nucleus of spermatocytes at prophase I of the meiotic cell cycle and was then found in the cytoplasm of round spermatids, revealing expression patterns of lncRNA during germ cell development. Collectively, the in situ hybridization method demonstrated in this study achieved the detection and comparison of precise distribution patterns of coding and non-coding RNAs at subcellular levels in single cells of adult tissues and organs.

**Conclusions:**

This high-sensitivity and high-resolution in situ hybridization is applicable to many vertebrate species and to various tissues and organs and will be useful for studies on the subcellular regulation of gene expression at the level of RNA localization.

**Electronic supplementary material:**

The online version of this article (10.1186/s12575-018-0071-z) contains supplementary material, which is available to authorized users.

## Background

Localization of mRNAs at subcellular regions plays fundamental roles in spatial and temporal gene expression in many organisms from yeast to mammals [1–5]. The standard methods for studying mRNA expression and localization in cells, tissues and organs are in situ hybridization techniques [6, 7]. Conventional in situ hybridization methods are generally based on probing each target mRNA with RNA probes labeled with biotin, digoxigenin (DIG) or fluorescein, followed by the detection of probes by specific antibodies and enzymatic reactions. However, detection of mRNA localization remains difficult because the expression levels of many mRNAs are low and the expression of such mRNAs in a single cell cannot be detected by conventional methods.

Amplification of signals in conventional in situ hybridization methods by using a tyramide signal amplification (TSA) system has facilitated the identification of mRNAs localized at specific subcellular regions in embryos and cultured cells [8–12]. Those studies revealed that thousands of mRNAs are localized at subcellular regions during embryogenesis and in third instar larval tissues in *Drosophila* [8, 9] and that all seven mRNAs examined are localized within the cytoplasm of cultured chicken cells [10]. In addition, cell biological and biochemical analyses have shown that approximately 50 mRNAs are localized in cell protrusions during migration of cultured mouse cells [13] and that 5–10% of mRNAs are localized to microtubules in *Xenopus* eggs and human cultured cells [14]. Accumulated evidence has shown that localization of mRNAs is a main regulatory mechanism of gene expression in cells. In contrast to *Drosophila* embryos and cultured mammalian cells which enable detection of mRNAs in whole-mount in situ hybridization, techniques for detection of mRNAs with high sensitivity and high resolution have not been established for vertebrate adult tissues and organs because of their large size, which prevents detection of mRNAs in deep layers when using distinct organs in whole.

In many tissues and organs of diverse species, huge numbers of non-coding RNAs (ncRNAs) are transcribed from their genomes [15–17]. ncRNAs longer than 200 nucleotides are called long non-coding RNAs (lncRNAs), and a number of them have been shown to function in various processes of gene expression including transcription, splicing and translational regulation [18–20]. Analyses of nuclear and subcellular localization of lncRNAs are important for understanding where, when and how individual lncRNAs function. A recent study using the TAS system in conventional in situ hybridization methods demonstrated that hundreds of lncRNAs are expressed in specific tissues and organs during *Drosophila* development and showed unique localization patterns within the cells, suggesting a wide range of functions of lncRNAs in various cellular processes [9]. However, detection of lncRNAs is still very difficult because the majority of lncRNAs are expressed at very low levels in a single cell [21].

A single-molecule fluorescent in situ hybridization (smFISH) method is based on probing each mRNA with 50–100 singly labeled oligonucleotide probes of length 20 bases and is applicable for detection of coding and non-coding RNAs with high-sensitivity in cultured cells and embryos [22, 23]. This technique can be combined with frozen and paraffin sections [24, 25]. However, preparation of 50–100 different oligonucleotides and labeling them with fluorophores are laborious and resource intensive, particularly for detecting a large number of different target mRNAs. RNAscope is a novel in situ hybridization method, which is based on a series of hybridization events with approximately 20 pairs of branched oligonucleotide probes and enables detection of mRNAs with high-sensitivity [26]. However, preparation of 20 pairs of branched oligonucleotide probes for target mRNAs is not inexpensive. Establishment of a method such as in situ hybridization with the TSA system combined with paraffin sections allows convenient and cost-effective preparation of probes, which are the same with those used in conventional in situ hybridization methods, and enables highly sensitive detection of RNAs by using differently labeled tyramide molecules in single cells at subcellular levels while preserving tissue and cell morphology.

In this study, we examined the expression and subcellular localization of mRNAs known to play important roles in oogenesis and embryogenesis. We first examined the expression of *Pou5f1/Oct4* mRNA in mouse ovaries by making paraffin sections and performing in situ hybridization using the TSA system. Although the expression of this transcript in the adult ovary remained obscure, we clearly showed *Pou5f1/Oct4* mRNA expression in mouse oocytes. We then examined the subcellular localization of *mos*, *cyclin B1* and *dazl* mRNAs in zebrafish ovaries. During zebrafish oogenesis, *mos*, *cyclin B1* and *dazl* mRNAs are accumulated as translationally repressed forms and are known to be localized at the animal or vegetal polar cytoplasm of the oocyte [27–35]. Double fluorescence in situ hybridization with the TSA system showed some similarities and many differences in distribution patterns of these mRNAs at subcellular levels in the oocyte cytoplasm. Our method also showed detailed distribution patterns of *Cyclin B1* and *Dazl* mRNAs in mouse ovaries. We further examined the expression patterns of *lncRNA-HSVIII* in mouse testes using our method. This lncRNA is transcribed from the genomic locus downstream of the *Prss42/Tessp-2* gene and shows a testis-specific expression pattern [36]. Our method clearly showed the nuclear distribution of this lncRNA in an early stage of spermatocytes and cytoplasmic distributions in later stages. Thus, our method enables detection of coding and non-coding RNAs in vertebrate tissues and organs with high-sensitivity and high-resolution while maintaining tissue and cell morphology in a cost-effective manner and can facilitate studies of the localization of RNAs at subcellular levels.

## Methods

### Isolation and Fixation of Ovaries and Testes

Zebrafish ovaries were dissected from adult females in zebrafish Ringer’s solution (116 mM NaCl, 2.9 mM KCl, 1.8 mM CaCl_2_, and 5 mM HEPES; pH 7.2). The ovaries were further dissected into several pieces (5–10 mm in diameter) with forceps under a SZ-ST dissecting microscope (Olympus). Mouse ovaries and testes were dissected from 8-week-old females and males in phosphate buffered saline (PBS: 137 mM NaCl, 2.7 mM KCl, 10 mM Na_2_HPO_4_, and 2 mM KH_2_PO_4_; pH 7.2). Ovaries of zebrafish and mice were fixed with 4% paraformaldehyde in PBS (4% PFA/PBS) for 3 h to overnight at 4 °C and then washed with PBS three times. Testes of mice were fixed with 4% PFA/PBS for 3 h at 4 °C and then immediately washed with PBS three times. Note that fixation of mouse testes for longer than 3 h at 4 °C results in severe shrinkage in testicular tubes and impaired tissue morphology (data not shown).

### Slide Coating

For paraffin sections, glass slides were coated with gelatin as follows. Gelatin powder (Wako Pure Chemical Industries, Ltd.) was dissolved in double distilled water (DDW) at 60 °C. Microscope glass slides (Matsunami Glass Industries, Ltd.) were incubated with 0.5% gelatin in DDW containing 0.05% CrK(SO_4_)_2_ for 15 min. Then the slides were dried and stored. Reagents used in this study are listed in Additional file 1: Table S1.

### Paraffin Sections

**Fig. 1:**
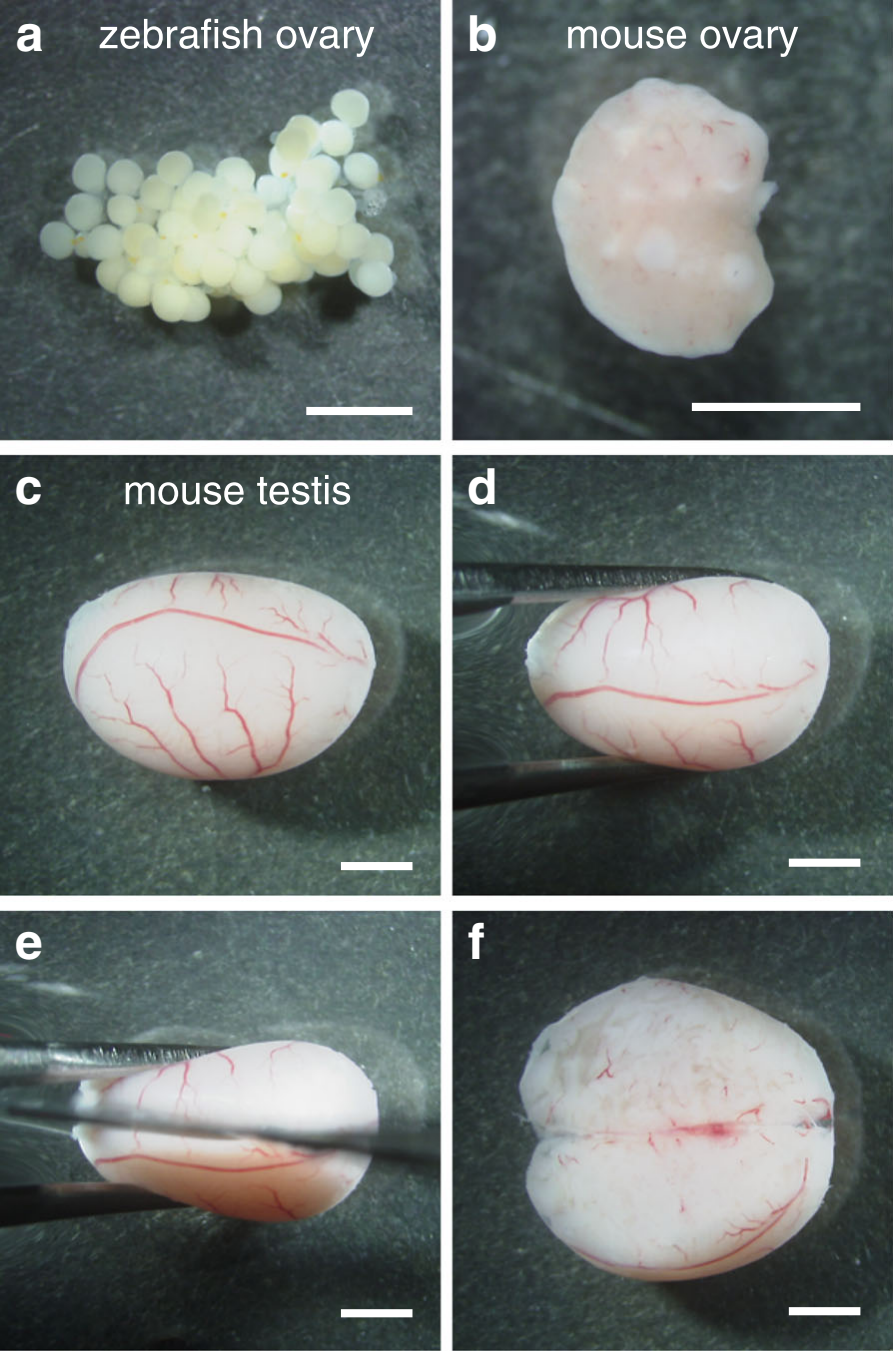
Photographs of zebrafish and mouse ovaries and testis. **a** A zebrafish ovary after being dissected into several pieces, followed by fixing with 4% PFA/PBS and washing with PBS. **b** A mouse ovary after being fixed with 4% PFA/PBS and washed with PBS. **c**-**f** A mouse testis after being fixed with 4% PFA/PBS and washed with PBS (**c**) that was held with forceps (**d**) and cut into two pieces with a razor blade according to the median plane (**e** and **f**). Bars: 2 mm

For paraffin embedding, the samples were dehydrated first with an amphipathic solvent and then with hydrophobic solvents as follows. Unless otherwise mentioned, incubation steps were performed at room temperature. Fixed ovaries of zebrafish (Fig. 1a) and mice (Fig. 1b) were dehydrated with 70%, 80%, 90%, 95%, and 99% ethanol each for 15 min with gentle shaking. Incubation with 99% ethanol was performed twice. Then the samples were incubated with isoamyl acetate (Wako Pure Chemical Industries, Ltd.) for 15 min with gentle shaking. This process was performed twice. Samples can be held for several days in this solvent. Before embedding, the samples were incubated with Lemosol (Wako Pure Chemical Industries, Ltd.) for 15 min with gentle shaking. This process was performed twice. Notably, incubation with isoamyl acetate makes samples to be easily cut into thin sections, particularly in the case of zebrafish oocytes, which contain large amounts of yolks. Alternatively, isoamyl acetate and Lemosol incubation can be replaced by incubation with xylene. Then the samples were placed in paraffin (Fisher Scientific; melting point, 56–57 °C) previously melted at about 60 °C in a dish and incubated at about 60 °C for 15 min on a NHP-1 hot plate (Iuchi). This process was repeated three times by transferring the samples to different dishes containing melted paraffin, using glass pipets or forceps. Finally, the samples were embedded in freshly prepared paraffin and immediately cooled on ice until the paraffin was completely set.

Each of the fixed mouse testes was cut into halves with a razor blade according to the median plane under the SZ-ST dissecting microscope (Fig. 1c-f). This process is important for complete dehydration of the testes. Dehydration of testes was performed according to the procedure described above with slight modifications. Testes of mice were incubated with ethanol, isoamyl acetate and Lemosol each for 45 min. Then the samples were incubated with paraffin for 45 min twice and finally incubated overnight with fresh paraffin. After incubation, the samples were embedded in freshly prepared paraffin.

The embedded zebrafish ovaries were cut into 5-μm-thick sections for detecting mRNA distribution in stage I oocytes and 9–12-μm-thick sections for detecting signals in later stage oocytes using a LR-85 microtome (Yamato Kohki Industrial Co., Ltd.). The embedded mouse ovaries were cut into 8–10-μm-thick sections, and the embedded mouse testes were cut into 7-μm-thick sections. The sections were floated on DDW prepared on gelatin-coated glass slides. The slides were then incubated on a HI1220 flattening table (Leica Biosystems) at 42 °C to flatten the sections until the slides were completely dried.

### Solution Preparation

For in situ hybridization, stock solutions were prepared as follows. 20X saline-sodium citrate (20X SSC: 3 M NaCl, 300 mM sodium citrate; pH 7.0) was autoclaved and stored. Torula RNA (Sigma) was dissolved in DDW at 50 °C and purified by phenol-, phenol-chloroform-, and chloroform-extraction. The RNA was precipitated, dissolved in DDW (10 mg/ml) and stored at − 20 °C. 20X Denhardt’s solution was prepared by dissolving Ficol-400 (0.4%), polyvinylpyrrolidone (0.4%) and bovine serum albumin (0.4%) in DDW and was stored at − 20 °C.

### Probe Preparation

DIG- and fluorescein-labeled RNA probes were prepared by in vitro transcription with RNA polymerases and plasmid vectors containing target transcript sequences. One μg of linearized plasmid DNAs was used as a template, and RNA probes were synthesized with SP6, T7 or T3 RNA polymerase (Roche) and DIG or Fluorescein RNA Labeling Mix (Roche) for 2–3 h at 37 °C. After precipitation with ethanol and lithium chloride, the RNA probes were dissolved in DDW. After determining the concentrations, the RNA probes were diluted (50 ng/μl) with probe dilution buffer (50% formamide, 5X SSC, 0.1% Tween-20) containing torula RNA (5 mg/ml) and were stored at − 20 °C.

In this study, we prepared 8 DIG-labeled antisense and sense RNA probes for the full lengths of mouse *Pou5f1/Oct4* and zebrafish *cyclin B1* gene transcripts and for the parts of zebrafish *dazl* and mouse *Dazl* gene transcripts (Additional file 1: Table S2). In addition, we prepared 6 fluorescein-labeled antisense and sense RNA probes for the full lengths of zebrafish *mos*, zebrafish *cyclin B1*, and mouse *Cyclin B1 *gene transcripts (Additional file 1: Table S2). Two DIG-labeled and 2 fluorescein-labeled antisense and sense RNA probes for the full length of *lncRNA-HSVIII* were also prepared (Additional file 1: Table S2). Sequences of the all transcripts used for making RNA probes were shown in Additional file 1: Table S3.

### Rehydration and Proteinase K Stimulation

The sections of ovaries and testes on glass slides were incubated with xylene for 5 min to remove paraffin. This process was performed twice. After removing xylene with 99% ethanol, the sections were rehydrated with 70% ethanol and PBS each for 2 min. The incubation with PBS was performed twice. When tissues of interest possess endogenous peroxidase activity, samples can be incubated with 1% H_2_O_2_ for 15–60 min to quench the activity. In this study, we did not quench the samples of ovaries and testes. Then the sections were treated with 1% Triton-X100 in PBS for 5 min. After washing with PBS twice, the sections were treated with 0.2 N HCl for 5 min. After washing again with PBS twice, the sections were incubated with 1 μg/ml Proteinase K (Sigma) in PBS at 37 °C for 5 min. The sections were washed again with PBS twice and fixed with 4% PFA/PBS for 5 min, followed by incubation with 100 mg/ml glycine in PBS for 15 min. The incubation with glycine was performed twice. Then the sections were incubated with a prehybridization buffer for sections (70% formamide, 2X SSC) for 60 min.

### Hybridization

The sections of ovaries and testes were hybridized with a mixture of 0.25–2 ng/μl of the fluorescein- and/or DIG-labeled RNA probes in hybridization mix solution (70% formamide, 20 mM Tris-HCl; pH 8.0, 2.5 mM EDTA, 1X Denhardt’s solution, 30 mM NaCl, 1 mg/ml torula RNA) containing 10% dextran sulfate at 45 °C overnight in a moisture chamber. During this incubation, the sections were covered with parafilm. The probe concentrations depended on the probes and expression levels of transcripts (see the Results section). After incubation, the parafilm was removed in 5X SSC at 50 °C. The sections were then washed with 50% formamide in 2X SSC at 50 °C for 30 min. After incubation with TNE (10 mM Tris, 500 mM NaCl, 1 mM EDTA; pH 7.5) at 37 °C for 10 min, the sections were incubated with 20 μg/ml RNase A (Sigma) in TNE at 37 °C for 30 min to reduce nonspecific background signals [7]. Then the sections were washed with TNE at 37 °C for 10 min, 2X SSC at 50 °C for 20 min, and 0.2X SSC at 50 °C for 20 min. The incubation with 0.2X SSC was performed twice. Then, the sections were incubated with TNT (100 mM Tris, 150 mM NaCl, 0.5% Tween-20; pH 7.5) at least for 5 min.

### Detection of DIG- and Fluorescein-Labeled RNA Probes

Single in situ hybridization of mRNA encoding *Pou5f1/Oct4* in mouse ovaries was performed as follows. After hybridization with the DIG-labeled *Pou5f1/Oct4* RNA probes as described above, the sections were blocked with a blocking buffer (0.5% blocking reagent (PerkinElmer, Inc), 100 mM Tris, 150 mM NaCl; pH 7.5) for 30 min in a moisture chamber. Then the sections were treated with anti-DIG-horseradish peroxidase (HRP) antibody (Roche) (1:500 dilution in blocking buffer) for 30 min in a moisture chamber. After washing with TNT three times, the sections were treated with tyramide-dinitrophenyl (DNP) (PerkinElmer, Inc.) (1:50 dilution in 1X Plus Amplification Diluent (PerkinElmer, Inc.), followed by dilution with an equal volume of DDW) for 20 min in a moisture chamber. After washing again with TNT three times, the samples were treated for 30 min with anti-DNP-alkaline phosphatase (AP) antibody (PerkinElmer, Inc.) (1:500 dilution in blocking buffer) in a moisture chamber. The samples were washed with TNT three times. After washing with a staining buffer (100 mM Tris, 100 mM NaCl, 50 mM MgCl_2_; pH 9.5), the samples were reacted with mixture of 225 μg/ml of nitro blue tetrazolium (NBT) and 175 μg/ml of 5-bromo-4-chloro-3-indolyl phosphate (BCIP) in staining buffer in a moisture chamber. The reaction was stopped with a stop solution (10 mM Tris, 1 mM EDTA; pH 8.0). The samples were then mounted with glycerol and observed under an Axioskop microscope (Carl Zeiss).

Double fluorescence in situ hybridization of mRNAs encoding Mos and Cyclin B1 in zebrafish ovaries was performed as follows. After hybridization with fluorescein-labeled *mos* RNA probes and DIG-labeled *cyclin B1* RNA probes, the sections were blocked with the blocking buffer for 30 min in a moisture chamber. The sections were then treated with anti-Fluorescein-HRP antibody (Roche) (1:200 dilution in blocking buffer) for 30 min in a moisture chamber. After washing with TNT three times, the sections were treated with tyramide-Cy3 (PerkinElmer, Inc.) (1:50 dilution in 1X Plus Amplification Diluent (PerkinElmer, Inc.), followed by dilution with an equal volume of DDW) for 20 min in a moisture chamber. The samples were washed again with TNT three times and then treated with 1% H_2_O_2_ in PBS for 15–60 min for inactivating HRP. When the amount of target transcripts was large and HRP could not be inactivated by this treatment, the samples were dehydrated with methanol, treated with 1% H_2_O_2_ in methanol for 30 min, and rehydrated with PBS. After washing with PBS twice, the sections were blocked with blocking buffer for 30 min in a moisture chamber. Then the samples were incubated with anti-DIG-HRP antibody (Roche) (1:500 dilution in blocking buffer) for 30 min in a moisture chamber. After washing with TNT three times, the sections were treated with tyramide-Fluorescein (PerkinElmer, Inc.) (1:50 dilution in 1X Plus Amplification Diluent, followed by dilution with an equal volume of DDW) for 20 min in a moisture chamber. The samples were then washed with TNT three times.

Double fluorescence in situ hybridization of mRNAs encoding Cyclin B1 and Dazl in zebrafish and mouse ovaries was performed as follows. After hybridization with fluorescein-labeled *cyclin B1* RNA probes and DIG-labeled *dazl* RNA probes, the sections were blocked with blocking buffer for 30 min in a moisture chamber. The sections were then treated with anti-DIG-HRP antibody (Roche) (1:500 dilution in blocking buffer) for 30 min in a moisture chamber. After washing with TNT three times, the sections were treated with tyramide-DNP (1:50 dilution in 1X Plus Amplification Diluent (PerkinElmer, Inc.), followed by dilution with an equal volume of DDW) for 20 min in a moisture chamber. After washing three times with TNT, the samples were treated overnight with anti-DNP-Alexa 488 antibody (Molecular Probes) (1:500 dilution in blocking buffer) in a moisture chamber. The samples were washed with TNT three times and then dehydrated with methanol, treated with 1% H_2_O_2_ in methanol for 30 min for inactivating HRP, and rehydrated with PBS. After washing with PBS twice, the sections were blocked with the blocking buffer for 30 min in a moisture chamber. Then the samples were incubated with anti-Fluorescein-HRP antibody (1:200 dilution in blocking buffer) for 30 min in a moisture chamber. After washing with TNT three times, the sections were treated with tyramide-Cy3 (1:50 dilution in 1X Plus Amplification Diluent, followed by dilution with an equal volume of DDW) for 20 min in a moisture chamber. The samples were then washed with TNT three times.

Single fluorescence in situ hybridization of *lncRNA-HSVIII* in mouse testes was performed as follows. After hybridization with fluorescein-labeled *lncRNA-HSVIII* RNA probes or DIG-labeled *lncRNA-HSVIII* RNA probes, the sections were blocked with the blocking buffer for 30 min in a moisture chamber. Then the sections were treated with anti-Fluorescein-HRP antibody (1:200 dilution in blocking buffer) or anti-DIG-HRP antibody (1:500 dilution in blocking buffer) overnight in a moisture chamber. After washing with TNT three times, the sections were treated with tyramide-Cy3 (1:50 dilution in 1X Plus Amplification Diluent, followed by dilution with an equal volume of DDW) for 20 min in a moisture chamber. The samples were then washed with TNT three times.

### Hoechst Staining and Mounting

To detect nuclei, the samples were incubated with 10 μg/ml Hoechst 33258 for 10 min. After being mounted with a Prolong Antifade Kit (Molecular probes) or Fluoro-KEEPER Antifade Reagent (Nacalai Tesque), the samples were observecd under a LSM5 LIVE confocal microscope (Carl Zeiss).

### Hematoxylin and Eosin (HE) Staining

To observe tissue and cell morphology, the sections of mouse ovaries and testes and zebrafish ovaries were stained with hematoxylin and eosin. After rehydration as described above, the sections were washed with running water for 5 min and incubated with hematoxylin staining solution for 2 min. After being washed with running water 30 min, the sections were incubated with eosin staining solution 30 s. The sections were then washed with running water 3 min, dehydrated with 70%, 80%, 90%, 95%, and 99% ethanol each for 1 min and incubated with xylene for 1 min. The samples were then mounted with a mounting medium MGK-S (Matsunami Glass Industries, Ltd.) and observed under the Axioskop microscope.

## Results

### Detection of *Pou5f1/Oct4* mRNA in the Mouse Ovary

**Fig. 2:**
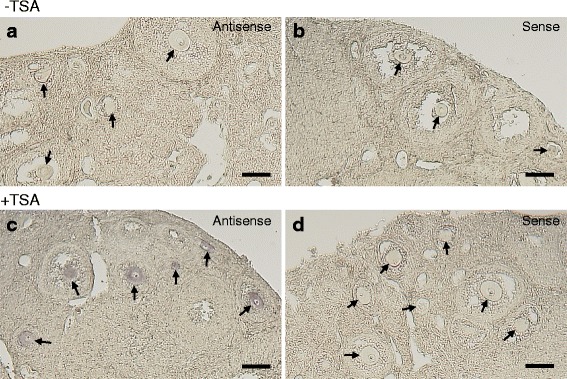
Single in situ hybridization of *Pou5f1/Oct4* mRNA (purple) in mouse ovaries. **a**-**b** Mouse ovary sections hybridized with the antisense (**a**) or sense (**b**) *Pou5f1/Oct4* RNA probe without amplification of signals using the TSA system (-TSA). No signal was detected in this conventional in situ hybridization method. **c**-**d** Mouse ovary sections hybridized with the antisense (**c**) or sense (**d**) *Pou5f1/Oct4* RNA probe with amplification of signals using the TSA system (+TSA). The signals were detected in oocytes of sections hybridized with the antisense RNA probe (**c**) but not in those hybridized with the sense RNA probe (**d**). Arrows indicate oocytes observed in the sections. Bars: 100 μm

Vertebrate oocytes of many species are arrested at prophase I of the meiotic cell cycle and accumulate large numbers of maternal factors including mRNAs during their growth. *Pou5f1/Oct4* mRNA encodes a transcriptional factor essential for mouse embryogenesis [37, 38]. This transcript is also necessary for maintenance or acquisition of pluripotency in embryonic stem (ES) cells or induced pluripotent stem (iPS) cells [39]. The expression of *Pou5f1/Oct4* mRNA is less abundant in oocytes [40] and has not been detected by in situ hybridization methods (Fig. 2a and b and data not shown). We examined the expression of *Pou5f1/Oct4* mRNA in mouse adult ovaries by making paraffin sections and performing in situ hybridization with or without the TSA system. Hybridization of 10 μm-thick mouse ovary sections with 1 ng/μl of the antisense *Pou5f1/Oct4* RNA probe showed no signal (Fig. 2a) as in the sections hybridized with 1 ng/μl of the sense *Pou5f1/Oct4* RNA probe (Fig. 2b) in a conventional in situ hybridization method without the TSA system. In contrast, the same hybridization procedure detected the expression of *Pou5f1/Oct4* mRNA in oocytes when the signals were amplified with the TSA system (Fig. 2c). Hybridization with the sense *Pou5f1/Oct4* RNA probe showed no signal (Fig. 2d). The tissue and cell morphology of mouse ovaries was observed by HE staining (Fig. 7a). Our method is highly reproducible because similar results were constantly obtained from three independent experiments (Additional file 1: Figure S1). These results indicate that in situ hybridization with the TSA system is highly sensitive and that *Pou5f1/Oct4* mRNA is specifically expressed in oocytes of mouse ovaries.

### Subcellular Localization of *mos* and *cyclin B1* mRNAs in the Zebrafish Ovary

We next examined the localization of *mos* and *cyclin B1* mRNAs simultaneously in zebrafish adult ovaries by making paraffin sections and performing double fluorescence in situ hybridization with the TSA system. *mos* mRNA encodes a kinase activating the mitogen-activated protein kinase (MAPK) pathway [41], and *cyclin B1* mRNA encodes a regulatory subunit of maturation/M-phase-promoting factor (MPF) [42]. These mRNAs are accumulated in the animal polar cytoplasm of zebrafish oocytes as a translationally repressed form [28, 29, 31, 32, 35, 43].

**Fig. 3:**
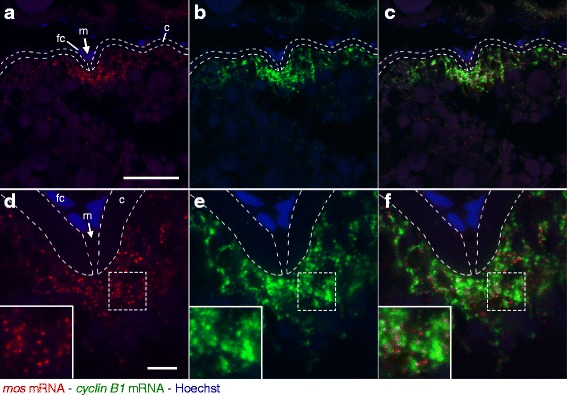
Double fluorescence in situ hybridization of *mos* (red) and *cyclin B1* (green) mRNAs in zebrafish ovaries. DNA is shown in blue. **a**-**c** A zebrafish ovary section showing localization of *mos* (**a**) and *cyclin B1* (**b**) mRNAs in a fully grown oocyte. A merged image is shown in (**c**). The *mos* and *cyclin B1* mRNAs were localized at the animal polar cytoplasm beneath the micropyle (m). fc, follicle cells; c, chorion. **d**-**f** High resolution imaging of the oocyte shown in **a**-**c**. The insets are enlarged views of the boxed regions. The *mos* and *cyclin B1* mRNAs were distributed in the animal polar cytoplasm of oocyte as different granules. Bars: 50 μm in **a**-**c**, 10 μm in **d**-**f**

Hybridization of 12 μm-thick zebrafish ovary sections with 1 ng/μl of the fluorescein-labeled antisense *mos* RNA probe and 0.25 ng/μl of the DIG-labeled antisense *cyclin B1* RNA probe, followed by amplification of signals using the TSA system, showed bright signals at the animal polar cytoplasm beneath the micropyle, a structure through which a sperm enters into the oocyte cytoplasm (Fig. 3a-c). In contrast, hybridization with the same concentrations of sense probes showed no signal (Additional file 1: Figure S2A-C). Since the expression level of *mos* mRNA is low, hybridization with low concentrations of the *mos* RNA probe (less than 1 ng/μl) resulted in low levels of signals. In contrast, a reduction in the concentration of *cyclin B1* RNA probe (less than 1 ng/μl) did not affect the levels of specific signals but reduced nonspecific signals probably due to the high levels of *cyclin B1* mRNA expression. In conclusion, the low concentration of the *cyclin B1* RNA probe (0.25 ng/μl) resulted in highly specific and less background signals. High resolution imaging of these two mRNAs showed that both mRNAs formed large granules in the animal polar cytoplasm of oocytes (Fig. 3d-f). However, the granules of *mos* mRNA were different from those of *cyclin B1* mRNA. The *mos* RNA granules barely co-localized with the *cyclin B1* RNA granules (3.9%, *n* = 282). Similar subcellular localization patterns of *mos* and *cyclin B1* mRNAs were observed in all oocytes of two independent experiments (Additional file 1: Figure S2D-F). The tissue and cell morphology of zebrafish ovaries was observed by HE staining (Fig. 7b-d). These results clearly showed the different distribution of *mos* and *cyclin B1* mRNAs although both mRNAs are localized at the same cytoplasmic regions. These results also showed the advantage of our method that enabled staining of distinct mRNAs without overlapping signals in the detection of distinct probes.

### Subcellular Localization of *cyclin B1* and *dazl* mRNAs in the Zebrafish Ovary

**Fig. 4:**
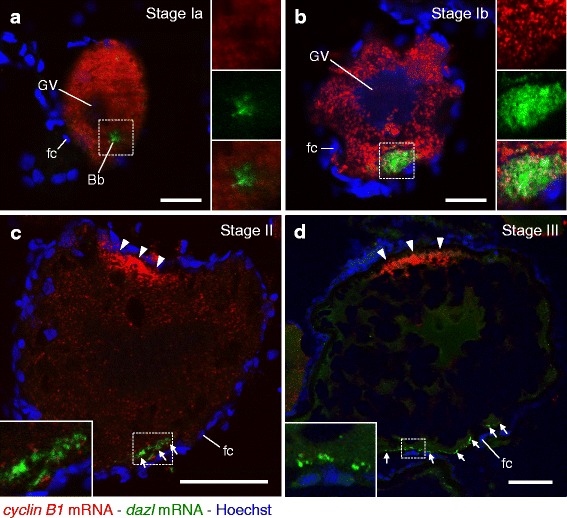
Double fluorescence in situ hybridization of *cyclin B1* (red) and *dazl* (green) mRNAs in zebrafish ovaries. DNA is shown in blue. **a** A follicle consisting of stage Ia oocyte. Insets are enlarged views of the boxed region showing *cyclin B1* mRNA (upper), *dazl* mRNA (middle) and a merged image (lower). **b** A follicle consisting of stage Ib oocyte. Insets are enlarged views of the boxed region showing *cyclin B1* mRNA (upper), *dazl* mRNA (middle) and a merged image (lower). **c** A follicle consisting of stage II oocyte. The inset is an enlarged view of the boxed region. **d** A follicle consisting of stage III oocyte. The inset is an enlarged view of the boxed region. Arrowheads indicate *cyclin B1* RNA granules localized at the animal polar cytoplasm of oocytes. Arrows indicate *dazl* RNA granules distributed in the vegetal polar cytoplasm of oocytes. GV, germinal vesicle; Bb, Balbiani body; fc, follicle cells. Bars: 20 μm in **a** and **b**, 50 μm in **c** and **d**

To further validate our method, we analyzed the expression of *cyclin B1* and *dazl* mRNAs in zebrafish oocytes. *dazl* mRNA encodes a zebrafish homolog of Deleted in Azoospermia (DAZ), an RNA-binding protein thought to be involved in primordial germ cell (PGC) development [33, 44, 45]. Hybridization of 5–12 μm-thick zebrafish ovary sections with 0.25 ng/μl of the fluorescein-labeled antisense *cyclin B1* RNA probe and 1 ng/μl of the DIG-labeled antisense *dazl* RNA probe, followed by amplification of signals using the TSA system, showed that *cyclin B1* mRNA was distributed throughout the cytoplasm except for the Balbiani body (Bb) marked by *dazl* mRNA in stage Ia oocytes (Fig. 4a). No obvious physiological structure of *cyclin B1* mRNA was observed in this stage. In contrast, *dazl* mRNA was localized at the Bb (Fig. 4a) as reported previously by using a conventional in situ hybridization method without the TSA system [34, 46]. The Bb is a universal and transient structure consisting of mitochondria, Golgi apparatus, endoplasmic reticulum, and certain RNAs [47]. In later stage (stage Ib) oocytes, the Bb is dispersed, leading to specification of the vegetal polar region and establishment of the animal-vegetal axis in oocytes [46, 48, 49]. *cyclin B1* mRNA was detected as granular structures throughout the cytoplasm of stage Ib oocytes (Fig. 4b, red signals). In this period, *dazl* mRNA moved to the vegetal cortex of oocytes (Fig. 4b, green signals). No obvious structure of *dazl* mRNA was observed in stage I oocytes. At stage II, *cyclin B1* mRNA becomes localized at the animal polar cytoplasm as persisting granular structures (Fig. 4c, arrowheads). In this stage, *dazl* mRNA was localized at the vegetal polar cytoplasm as granular structures (Fig. 4c, arrows). Large numbers of *cyclin B1* RNA granules were localized at the animal polar cytoplasm in stage III (Fig. 4d) and fully grown, stage IV oocytes [31, 32, 50]. The localization and granular structure of *dazl* mRNA were maintained in stage III and IV oocytes, but the granules were broadly distributed in the vegetal polar cytoplasm (Fig. 4d, arrows). In all stages, no signal was detected with the control sense probes (Additional file 1: Figure S3A-D). Similar distribution patterns of *mos* and *dazl* mRNAs during oocyte development were obtained from three independent experiments (Additional file 1: Figure S3E-H).

Double staining of *cyclin B1* and *dazl* mRNAs showed interesting aspects of the mRNA distribution and localization in oocytes. First, *cyclin B1* and *dazl* mRNAs were distributed in completely different regions in the cytoplasm of stage Ia oocytes (Fig. 4a) and localized at different poles in later stages (Fig. 4c and d). Second, in the period of Bb dispersion, *cyclin B1* mRNA was distributed in the region where the Bb was localized and was partially co-localized with *dazl* mRNA in this region (Fig. 4b, see insets). In contrast, *dazl* mRNA was not dispersed throughout the cytoplasm and maintained its aggregation while moving to the vegetal cortex (Fig. 4b). Finally, *cyclin B1* mRNA seemed to form granular structures from stage Ib (Fig. 4b), while *dazl* mRNA seemed to form such structures from stage II (Fig. 4c).

### Subcellular Localization of *Cyclin B1* and *Dazl* mRNAs in the Mouse Ovary

**Fig. 5:**
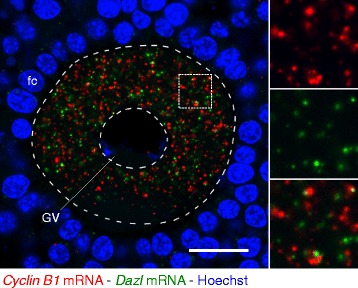
Double fluorescence in situ hybridization of *Cyclin B1* (red) and *Dazl* (green) mRNAs in mouse ovaries. DNA is shown in blue. Insets are enlarged views of the boxed region showing *Cyclin B1* mRNA (upper), *Dazl* mRNA (middle) and a merged image (lower). GV, germinal vesicle; fc, follicle cells. Bar: 20 μm

We next examined the distribution and localization of *Cyclin B1* and *Dazl* mRNAs in mouse adult ovaries. Hybridization of 8–10 μm-thick mouse ovary sections with 1 ng/μl of the fluorescein-labeled antisense *Cyclin B1* RNA probe and 1 ng/μl of the DIG-labeled antisense *Dazl* RNA probe, followed by amplification of signals using the TSA system, showed that *Cyclin B1* and *Dazl* mRNAs were distributed as granules in the cytoplasm of fully grown mouse oocytes (Fig. 5), as was observed for the zebrafish. However, in contrast to the zebrafish, *Cyclin B1* and *Dazl* RNA granules were not localized in different regions but were similarly distributed in the cytoplasm. Interestingly, *Cyclin B1* and *Dazl* mRNAs were found to be assembled into different granules, and these granules were distributed close to each other (Fig. 5, see insets). No signal was detected with the control sense probes (Additional file 1: Figure S4A). Similar distribution patterns of *Cyclin B1* and *Dazl* mRNAs were obtained from three independent experiments (Additional file 1: Figure S4B and C). Our method thus showed similarity and difference of the two mRNAs in distribution pattern and region-specific localization in zebrafish and mouse oocytes.

### Detection and Subcellular Localization of *lncRNA-HSVIII* in the Mouse Testis

We applied our method to detection of lncRNAs in the mouse testis. Thousands of lncRNAs have been detected in mammalian testes [51–56]. However, their expression patterns and subcellular distributions in testes have remained almost completely unknown. *lncRNA-HSVIII* showed a testis-specific expression pattern [36] and is thought to function in testis development and spermatogenesis. As in the case of other lncRNAs, *lncRNA-HSVIII* is a low-abundance RNA because qRT-PCR analysis showed that the expression level of *lncRNA-HSVIII* was approximately 770 times less than that of house-keeping *Aip* gene transcript [57], which encodes aryl hydrocarbon receptor interacting protein, in mouse testis (769.5 ± 129.1, *n* = 3).

**Fig. 6:**
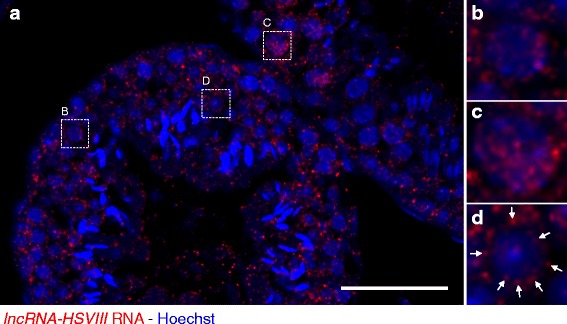
Fluorescence in situ hybridization of *lncRNA-HSVIII* (red) in mouse testes. DNA is shown in blue. **a** A transverse section of seminiferous tubules. **b** An enlarged view of the boxed region marked as B in a. **c** An enlarged view of the boxed region marked as C in a. **d** An enlarged view of the boxed region marked as D in a. Arrows indicate *lncRNA-HSVIII* distributed in the cytoplasm of round spermatid. Bar: 50 μm

**Fig. 7:**
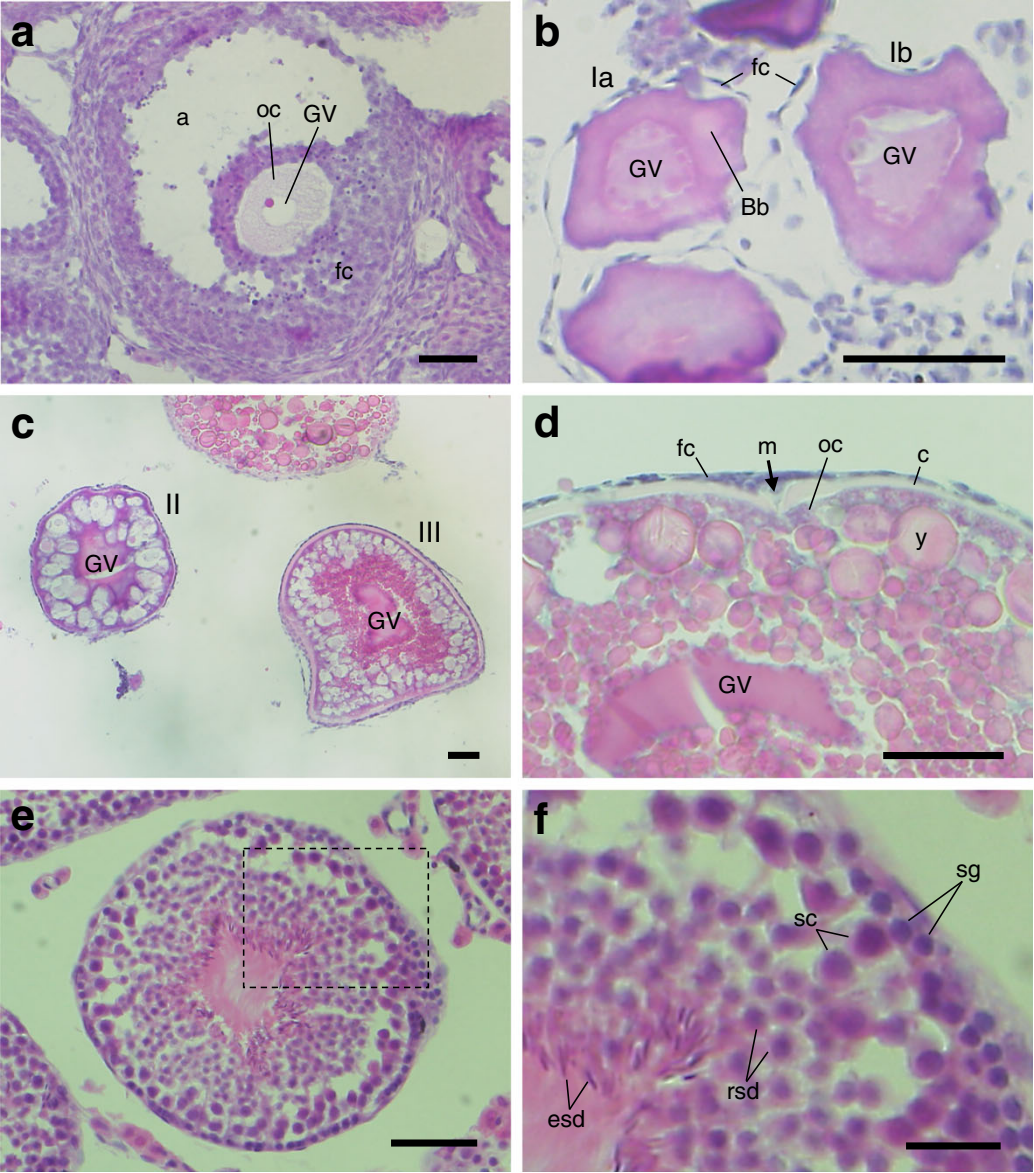
The tissue and cell morphology of mouse and zebrafish ovaries and mouse testes. **a** A section of mouse ovary stained with hematoxylin and eosin (HE). A follicle consisting of fully grown oocyte is shown. a, antrum; oc, oocyte cytoplasm; GV, germinal vesicle; fc, follicle cells. **b**-**d** Sections of zebrafish ovary stained with HE. Follicles consisting of stage I oocytes (**b**), stage II and III oocytes (**c**), and stage IV oocyte (**d**). GV, germinal vesicle; fc, follicle cells; Bb, Balbiani body; m, micropyle; oc, oocyte cytoplasm; c, chorion; y, yolk. **e** A transverse section of seminiferous tubules of mouse testis stained with HE. **f** An enlarged view of the boxed region marked in e. sg, spermatogonia; sc, spermatocytes; rsd, round spermatids; esd, elongating spermatids. Bars: 50 μm in A-E, 10 μm in F

We first examined the expression of *lncRNA-HSVIII* by making paraffin sections and performing in situ hybridization without amplification of signals using the TSA system. Unfortunately, no signal was detected by this method. We then examined the expression patterns of this lncRNA by combining paraffin sections and in situ hybridization with the TSA system. Hybridization of 7 μm-thick mouse testis sections with 2 ng/μl of the fluorescein-labeled antisense *lncRNA-HSVIII* RNA probe, followed by amplification of signals using the TSA system, showed that *lncRNA-HSVIII* were distributed as some dot-like signals in nuclei of spermatocytes at the early phase of prophase I of the meiotic cell cycle (Fig. 6a and b). The number of these foci increased in cells at the different phase of prophase I (Fig. 6a and c). The nuclear distribution of *lncRNA-HSVIII* disappeared, and, instead, this lnRNA was found to be distributed in the cytoplasm of round spermatids (Fig. 6a and d, arrows). In contrast, hybridization with the same concentration of sense probe showed slight but not specific signals, showing fluorescence background in this procedure (Additional file 1: Figure S5A). Furthermore, our method is highly reproducible in the detection of lncRNA as in the case of mRNA because similar expression and distribution patterns of *lncRNA-HSVIII* were obtained from four independent experiments using both the fluorescein- and DIG-labeled antisense *lncRNA-HSVIII* RNA probes (Additional file 1: Figure S5B-F). The tissue and cell morphology of mouse testis was observed by HE staining (Fig. 7e and f). Taken together, our method revealed the expression patterns of *lncRNA-HSVIII* in germ cells in testes.

## Discussion

### Detection and Subcellular Localization of mRNAs in Adult Tissues and Organs

Because of the limitations of techniques, the detection and analysis of subcellular localization of mRNAs and lncRNAs remain difficult. In this study, we produced 18 different RNA probes for the detection of mRNAs and lncRNA and showed the expression and subcellular localization of distinct RNAs in mouse and zebrafish adult ovaries and testes. Our method detects the mRNAs with high-sensitivity because 1) *Pou5f1/Oct4* mRNA could be detected in mouse oocytes (Fig. 2), which was not detected by a conventional method , and 2) *lncRNA-HSVIII* could be detected in mouse spermatocytes (Fig. 6), which was also undetectable in a conventional method. In addition, our method provides high-resolution imaging while maintaining tissue and cell morphology and is useful for simultaneous detection of two different mRNAs at subcellular levels. Double staining of *mos* and *cyclin B1* mRNAs in zebrafish oocytes demonstrated the distributions of these mRNAs as different granules even both mRNAs were localized in the same regions (Fig. 3). Since one can make biotin- or DNP-labeled RNA probes for detection of mRNAs of interest, the expression and localization of three and four mRNAs would be simultaneously analyzed by this method.

A previous study showed that zebrafish *cyclin B1* mRNA is initially distributed throughout the cytoplasm of oocytes (stages I and II) and becomes localized at the animal polar cytoplasm after stage III, when DIG-labeled RNA probes were recognized by an AP-coupled anti-DIG antibody, followed by detection of signals by precipitation of AP substrates [28]. Fluorescence in situ hybridization with the TSA system has revealed the localization of *cyclin B1* mRNA at the animal polar cytoplasm as RNA granules [31, 32, 50], and this study revealed changes in *cyclin B1* mRNA distribution and localization during oocyte development; i.e., *cyclin B1* mRNA was initially distributed throughout the cytoplasm of oocytes in stage I and subsequently moved to the animal pole in stage II (Fig. 4a-c), and a granular structure was first found in stage Ib oocytes and persisted in later stages (Fig. 4b-d). An explanation for the difference in distribution of *cyclin B1* mRNA in stage II oocytes is that precipitation of AP substrates tends to be diffused throughout the cytoplasm, while tyramide is associated with peptides in close proximity to POD-coupled antibodies. This difference was also demonstrated in the case of mRNAs expressed in *Drosophila* embryos [8]. Collectively, our method enables detection of the precise subcellular localization of mRNAs in vertebrate adult tissues and organs.

The localization of *dazl* mRNA during oogenesis in the zebrafish has been shown in previous studies in which the signals of RNA probes were detected by precipitation of AP substrates [34, 46]. Our fluorescence in situ hybridization with the TSA system showed similar localization patterns of *dazl* mRNA (Fig. 4). However, details of the distribution pattern were different. Previous studies showed that *dazl* mRNA was diffusely localized in the vegetal polar cytoplasm of later stage oocytes, probably due to diffusion of AP substrate precipitation in this region [33, 34, 46]. Our method revealed that *dazl* mRNA forms granular structures at the vegetal pole, and these granules were broadly distributed in the vegetal cytoplasmic region of later stage oocytes (Fig. 4c and d). This finding demonstrates that *dazl* mRNA does not diffusely distribute throughout the vegetal polar cytoplasm as single RNA-protein complexes but assembles into large structures in this region. Our observations will contribute to elucidation of the regulation of *dazl* mRNA during oocyte development and after fertilization.

Detection of *Cyclin B1* and *Dazl* mRNAs in mouse oocytes is difficult due to their low expression levels in a single oocyte. Our previous study showed the distribution of *Cyclin B1* mRNA in the mouse oocyte cytoplasm as granules by single fluorescence in situ hybridization with the TSA system [31]. In this study, double fluorescence in situ hybridization of *Cyclin B1* and *Dazl* mRNAs revealed that these two mRNAs were assembled into different granules and that these granules were distributed in similar regions in fully grown oocytes (Fig. 5). These results demonstrate for the first time the different subcellular distributions of mRNAs in the cytoplasm of mammalian oocytes.

### Conserved and Species-Specific Regulation of mRNAs in Zebrafish and Mouse Oocytes

Our results demonstrated similarity and difference of the mRNA regulations in zebrafish and mouse species. Granular structures of mRNAs have been suggested to function in regulating the timing of translational activation of the mRNAs [31, 32]. mRNAs encoding Cyclin B1 and Dazl appeared to form granular structures in both zebrafish and mouse oocytes, suggesting translational regulation of these mRNAs.

In contrast to the conservation in physiological structure, localization of these mRNAs appeared to be different in the zebrafish and mouse. This may be due to differences in the functions of Dazl protein. During zebrafish embryogenesis, maternally provided *dazl* mRNA and its protein product have been suggested to function in primordial germ cell (PGC) development [45] as in the case of *Xenopus* [44]. Localization of *dazl* mRNA at the vegetal polar cytoplasm of oocytes is one of the important processes for PGC development after fertilization [34, 45]. Although the zygotically expressed mouse *Dazl* gene has been shown to function in spermatogenesis [58, 59], maternally provided *Dazl* mRNA and its protein product might function in oocyte maturation but not in embryogenesis. A recent study has shown that translation of *Dazl* mRNA is activated at the early period of oocyte maturation, which is similar to the period of translational activation of *Cyclin B1* mRNA [60]. Synthesized Cyclin B1 protein activates MPF, and MPF promotes oocyte maturation by phosphorylating substrates of the catalytic subunit of Cdc2 [61–63], while synthesized Dazl protein promotes translational activation of other mRNAs, protein products of which are essential for promoting progression of oocyte maturation [60, 64]. Distributions of *Cyclin B1* and *Dazl* mRNAs in similar regions in mouse oocytes might be important for translational activation of these mRNAs at a similar timing.

### Detection and Subcellular Localization of lncRNAs in Adult Tissues and Organs

Genetic and functional analyses of lncRNAs have shown fundamental roles of this type of ncRNA in various cellular processes [17–20]. Some lncRNAs have been shown to act as structural and functional scaffolds for specialized subnuclear domains [65]. Other lncRNAs have been shown to participate in epigenetic regulation by interacting with chromatin-modifying complexes or to regulate post-transcriptional processes including alternative splicing, mRNA stability and translation [20, 66]. Systematic and comprehensive studies have demonstrated that thousands of lncRNAs are expressed in many different cell types and organs [67, 68], but their expression patterns in cells remain largely unknown. Our method revealed the nuclear distribution of *lncRNA-HSVIII* as some foci in spermatocytes at an early phase of prophase I and the cytoplasmic localization of this lncRNA in round spermatids (Fig. 4). Although it remains to be determined whether *lncRNA-HSVIII* functions in testis development and spermatogenesis, our findings suggest structural or regulatory functions of *lncRNA-HSVIII* in the nucleus of spermatocytes in early meiotic stages and post-transcriptional functions such as translational regulation in the cytoplasm of round spermatids. Intriguingly, a large-scale evolutionary study has demonstrated that more than 40% of lncRNAs show a testes-specific expression pattern in 11 species from frog to human, suggesting the importance of lncRNAs in spermatogenesis [69]. Our method has provided a new mode of localization patterns of lncRNAs during spermatogenesis and is useful for the future studies of lncRNAs in testes. Our method also enables determination of cell- and tissue-specific localization of lncRNAs in other organs, which may lead to a better understanding of their function.

## Conclusion

We have described a high-sensitivity and high-resolution in situ hybridization method that enables detection of the distribution and localization of RNAs at subcellular levels in large organs such as the ovary and testis. This method is applicable to many organisms and various tissues and organs and should facilitate studies of gene expression at the level of RNA regulation, which is functionally and mechanistically important for promoting various biological processes in diverse species.

## Additional file

Additional file 1: **Table S1.** Reagents used in this study. **Table S2.** RNA probes used for detection of target mRNAs. **Table S3.** Sequences of transcripts used for making RNA probes in this study. **Figure S1.** Single in situ hybridization of *Pou5f1/Oct4* mRNA in mouse ovaries. (**A**-**D**) Sections hybridized with the sense (**A** and **C**) or antisense (**B** and **D**) *Pou5f1/Oct4* RNA probe with amplification of signals using the TSA system (+TSA). Bars: 100 *μ*m. **Figure S2.** Double fluorescence in situ hybridization of *mos* and *cyclin B1* mRNAs in zebrafish ovaries. (**A**-**C**) A section hybridized with the sense probes for *mos* and *cyclin B1* mRNAs in a fully grown oocyte. (**D**-**F**) A section hybridized with the antisense probes for *mos* and *cyclin B1* mRNAs in a fully grown oocyte. Bars: 50 *μ*m in **A**-**C**, 10 *μ*m in **D**-**F**. **Figure S3.** Double fluorescence in situ hybridization of *cyclin B1* and *dazl* mRNAs in zebrafish ovaries. (**A**-**D**) Sections hybridized with the sense probes for *cyclin B1* and *dazl* mRNAs. Follicles consisting of stage Ia (**A**), stage Ib (**B**), stage II (**C**), and stage III (**D**) oocytes. (**E**-**H**) Sections hybridized with the antisense probes for *cyclin B1* and *dazl* mRNAs. Follicles consisting of stage Ia (**E**), stage Ib (**F**), stage II (**G**), and stage III (**H**) oocytes. Bars: 20 *μ*m in **A**, **B**, **E** and **F**, 50 μm in **C**, **D**, **G** and **H**. **Figure S4.** Double fluorescence *in situ* hybridization of *Cyclin B1* and *Dazl* mRNAs in mouse ovaries. (**A**) A section hybridized with the sense probes for *Cyclin B1* and *Dazl* mRNAs. (**B** and **C**) Sections hybridized with the antisense probes for *Cyclin B1* and *Dazl* mRNAs. Bars: 20 *μ*m. **Figure S5.** Fluorescence *in situ* hybridization of *lncRNA-HSVIII* in mouse testes. (**A**-**B**) Transverse sections of seminiferous tubules hybridized with the fluorescein-labeled sense (**A**) and antisense (**B**) probes. (**C**-**F**) Transverse sections of seminiferous tubules hybridized with the DIG-labeled sense (**C** and **E**) and antisense (**D** and **F**) probes. Bars: 50 *μ*m. (PDF 16.6 mb)
