# Supplementary material for: High-Sensitivity and High-Resolution In Situ Hybridization of Coding and Long Non-coding RNAs in Vertebrate Ovaries and Testes
Source: Biol Proced Online. 2018 Mar 1;20:6. doi: 10.1186/s12575-018-0071-z (PMC5831722; doi:10.1186/s12575-018-0071-z)
Supplement: Supplementary file 1 — Table S1. Reagents used in this study. Table S2. RNA probes used for detection of target mRNAs. Table S3. Sequences of transcripts used for making RNA probes in this study. Figure S1. Single in situ hybridization of Pou5f1/Oct4 mRNA in mouse ovaries. (A-D) Sections hybridized with the sense (A and C) or antisense (B and D) Pou5f1/Oct4 RNA probe with amplification of signals using the TSA system (+TSA). Bars: 100 μm. Figure S2. Double fluorescence in situ hybridization of mos and cyclin B1 mRNAs in zebrafish ovaries. (A-C) A section hybridized with the sense probes for mos and cyclin B1 mRNAs in a fully grown oocyte. (D-F) A section hybridized with the antisense probes for mos and cyclin B1 mRNAs in a fully grown oocyte. Bars: 50 μm in A-C, 10 μm in D-F. Figure S3. Double fluorescence in situ hybridization of cyclin B1 and dazl mRNAs in zebrafish ovaries. (A-D) Sections hybridized with the sense probes for cyclin B1 and dazl mRNAs. Follicles consisting of stage Ia (A), stage Ib (B), stage II (C), and stage III (D) oocytes. (E-H) Sections hybridized with the antisense probes for cyclin B1 and dazl mRNAs. Follicles consisting of stage Ia (E), stage Ib (F), stage II (G), and stage III (H) oocytes. Bars: 20 μm in A, B, E and F, 50 μm in C, D, G and H. Figure S4. Double fluorescence in situ hybridization of Cyclin B1 and Dazl mRNAs in mouse ovaries. (A) A section hybridized with the sense probes for Cyclin B1 and Dazl mRNAs. (B and C) Sections hybridized with the antisense probes for Cyclin B1 and Dazl mRNAs. Bars: 20 μm. Figure S5. Fluorescence in situ hybridization of lncRNA-HSVIII in mouse testes. (A-B) Transverse sections of seminiferous tubules hybridized with the fluorescein-labeled sense (A) and antisense (B) probes. (C-F) Transverse sections of seminiferous tubules hybridized with the DIG-labeled sense (C and E) and antisense (D and F) probes. Bars: 50 μm. (PDF 16.6 mb) [file 12575_2018_71_MOESM1_ESM.pdf]

Table S1. Reagents used in this study.

Slide coating
- Gelatin powder (Wako Pure Chemical Industries, Ltd., cat.no. 077-03155)
Paraffin sections
- Isoamyl acetate (Wako Pure Chemical Industries, Ltd., cat.no. 016-03646)
- Lemosol (Wako Pure Chemical Industries, Ltd., cat.no. 128-03993)
- Paraffin (Fisher Scientific; melting point, 56-57°C, cat.no. T-565-1)
Stock solutions
- Torula RNA (Sigma, cat.no. R6625)
Probe preparation
- SP6 RNA polymerase (Roche, cat.no. 810 274)
- T7 RNA polymerase (Roche, cat.no. 881 767)
- T3 RNA polymerase (Roche, cat.no. 1 031 163)
- DIG RNA Labeling Mix (Roche, cat.no. 1 277 073)
- Fluorescein RNA Labeling Mix (Roche, cat.no. 1 685 619)
Proteinase K stimulation
- Proteinase K (Sigma, cat.no. P2308)
Hybridization
- RNase A (Sigma, cat.no. R4875)
Detection of DIG- and fluorescein-labeled RNA probes
- Blocking reagent (PerkinElmer, Inc., cat.no. FP1020)
- Anti-DIG-HRP antibody (Roche, cat.no. 1 207 733)
- Anti-Fluorescein-HRP antibody (Roche, cat.no. 1 426 346)
- Anti-DNP-AP antibody (PerkinElmer, Inc., cat.no. NEL746A)
- Anti-DNP-Alexa 488 antibody (Molecular Probes, cat.no. A-11097)
- Tyramide-DNP (PerkinElmer, Inc., cat.no. NEL746A)
- Tyramide-Cy3 (PerkinElmer, Inc., cat.no. NEL744)
- Tyramide-Fluorescein (PerkinElmer, Inc., cat.no. NEL741)
Mounting
- Prolong Antifade Kit (Molecular probes, cat.no. P7184)
- Fluoro-KEEPER Antifade Reagent (Nacalai Tesque, cat.no. 12593-64)

Table S2. RNA probes used for detection of target mRNAs.

Species	Transcripts	Labeling	Strands
Mouse	*Pou5f1/Oct4*	DIG	Antisense/Sense
Zebrafish	*mos*	Fluorescein	Antisense/Sense
Zebrafish	*cyclin B1*	DIG, Fluorescein	Antisense/Sense
Zebrafish	*dazl*	DIG	Antisense/Sense
Mouse	*Cyclin B1*	Fluorescein	Antisense/Sense
Mouse	*Dazl*	DIG	Antisense/Sense
Mouse	*lncRNA-HSVIII*	DIG, Fluorescein	Antisense/Sense

Table S3. Sequences of transcripts used for making RNA probes in this study.

Mouse *Pou5f1/Oct4*

GAGGTGAAACCGTCCCTAGGTGAGCCGTCTTTCCACCAGGCCCCCGGCTCGGGGTGCCCA
CCTTCCCCATGGCTGGACACCTGGCTTCAGACTTCGCCTTCTCACCCCCACCAGGTGGGG
GTGATGGGTCAGCAGGGCTGGAGCCGGGCTGGGTGGATCCTCGAACCTGGCTAAGCTTCC
AAGGGCCTCCAGGTGGGCCTGGAATCGGACCAGGCTCAGAGGTATTGGGGATCTCCCCAT
GTCCGCCCGCATACGAGTTCTGCGGAGGGATGGCATACTGTGGACCTCAGGTTGGACTGG
GCCTAGTCCCCCAAGTTGGCGTGGAGACTTTGCAGCCTGAGGGCCAGGCAGGAGCACGAG
TGGAAAGCAACTCAGAGGGAACCTCCTCTGAGCCCTGTGCCGACCGCCCCAATGCCGTGA
AGTTGGAGAAGGTGGAACCAACTCCCGAGGAGTCCCAGGACATGAAAGCCCTGCAGAAGG
AGCTAGAACAGTTTGCCAAGCTGCTGAAGCAGAAGAGGATCACCTTGGGGTACACCCAGG
CCGACGTGGGGCTCACCCTGGGCGTTCTCTTTGGAAAGGTGTTCAGCCAGACCACCATCT
GTCGCTTCGAGGCCTTGCAGCTCAGCCTTAAGAACATGTGTAAGCTGCGGCCCCTGCTGG
AGAAGTGGGTGGAGGAAGCCGACAACAATGAGAACCTTCAGGAGATATGCAAATCGGAGA
CCCTGGTGCAGGCCCGGAAGAGAAAGCGAACTAGCATTGAGAACCGTGTGAGGTGGAGTC
TGGAGACCATGTTTCTGAAGTGCCCGAAGCCCTCCCTACAGCAGATCACTCACATCGCCA
ATCAGCTTGGGCTAGAGAAGGATGTGGTTCGAGTATGGTTCTGTAACCGGCGCCAGAAGG
GCAAAAGATCAAGTATTGAGTATTCCCAACGAGAAGAGTATGAGGCTACAGGGACACCTT
TCCCAGGGGGGGCTGTATCCTTTCCTCTGCCCCCAGGTCCCCACTTTGGCACCCCAGGCT
ATGGAAGCCCCCACTTCACCACACTCTACTCAGTCCCTTTTCCTGAGGGCGAGGCCTTTC
CCTCTGTTCCCGTCACTGCTCTGGGCTCTCCCATGCATTCAAACTGAGGCACCAGCCCTC
CCTGGGGATGCTGTGAGCCAAGGCAAGGGAGGTAGACAAGAGAACCTGGAGCTTTGGGGT
TAAATTCTTTTACTGAGGAGGGATTAAAAGCACAACAGGGGTGGGGGGTGGATGGGGAA
AGAAGCTCAGTGATGCTGTTGATCAGGAGCCTGGCCTGTCTGTCACTCATCATTTTGTTC
TTAAATAAAGACTGGGACACACAGTAGATAGCT

Zebrafish *mos*

AAATTAGCGACCAGCTCTCCGATTTCACAGTTATCATGCGCCATGCCCTCACCAATCCCC
GTCACCCGACTTTTGCCAAAGGATTTCGGCCTCGAGTTTGGCGCATGCAGCAGCCCGCTG
ACCAAAACTGCCAGTGGATCTACCCTGCGCGTGCCCACAAACAAGTTTCATGGTAAAGTC
GCACACAGGCTCTGGTCCTCCGTGATCCACTGGCGCGAGCTGCAGGCTCTGGAGCCCATA
GGCAGCGGTGGATTCGGTACGGTGTTCAGAGGCACATACTTCGGCGAGACTGTCGCTGTG
AAAAAGGTCAAGTGTGTGAAAAACAAACTGGCATCGAGGCAAAGTTTCTGGGCGGAACTC
AACGCCGCGCACCTGCACCATCAAAACATTGTGCGCGTGCTCGCGGCCACCACGTGCACT
CCTGCGCATCTCAACACCAAAGACAACATCGGGACGATCGTAATGGAGTTCGCAGGCAAT
ATAAATCTACAGAAGCTCATTTATGGGCTCACAGACTTGCTTCCTGTGGAGAAGTGTATA
AAGTATTCAATAGACATCGCGCGCGCCCTCCAGCACCTGCACGCGCACGGCGTAGTGCAC
CTGGATTTAAAACCAGCCAATGTCTTGTTGTCAGAACAGGGTGTTTGTAAAATCGCAGAT
TTTGGGTGCTCGTTTAAAATATCCAGCACAAGTGACACCGTGACGCACATGAATGAAATC
GGCGGCACGTTTACGCACCGGGCGCCCGAGCTGCTGAAAGGTGAGGAAGTGTCGCCGCGC
GTGGACGTTTATTCTTTTGGCATAACGTTGTGGCAGCTGCTCACCCGAGAGCCGCCCTAT
GAGGGAGACAGACAGTATATCCTGTACGCTGTTGTGGGGTATAACCTGCGCCCTTTGACC
AGCAGGAATGTTTTTACCCAGTTTTTTATTGGACAGAATTGTCAAAAACTGATCAGCCGG
TGTTGGGACGGCGACCCCAGCATCCGACCGACCGCAGATAAGTTTGTCGACGAACTTTCA
GTTTTACTGTAAAATTGAATCTCCGCGTTTGCAAAAAAAAAAAAAAAAAAAAATCTTCAAT
GAAACGTTTTTTTATAACAATTGTTTAATATTGTAAATGTTCGTGTTTTTGTTTTATTGT
GAAGCTAAATTTTTTATGCAAAAATGTTTAATTAAATGTTTCCTTGTGTTT

Zebrafish *cyclin B1*

GGCACGAGTAAACCTCTGTAGAGAAATAGTGCTATATTGAGCTCGCTTGAGTAGATTTTA
CCAGTTTGTTCATCGAGTCACAGCAATAAACCACGAGAAATGATGGCTCTCCGTGTCACA
AGGAACACTCGCCTGGCCAGCAGCGAGAATCAGAACGCTCTGCCCGGAAAAGCAGTTGTA
GCGAACAAGCCCGGACTCAGACCAAGGGCCGCGCTGGGGGAGATTGGCAACAATCCGCAA
ACACGACAGGCTTTGAAGAAGAAGGAGGTGAAGGTTGCACCCGCCGCCGAGGTTGTGGTT
GAGAAGGCACCTGTGGTTCAACAGCCCAAGAAGGATTCTCCTAAGGTTCAACATGGCGTT
AAGGTTGTGTCTGAGCCTTCCTCTCCTGTTCCTATGGAGACCTCCGGCTGTGCTTCAGAT
GATCTGTGTCAAGCATTCTCTGATGTTCTGCTTAATATCAAGGATGTTGACGCAGATGAT
TATGATAATCCCATGCTTTGCAGTGAATATGTCAAGGACATCTATTTGTATTTGCGCCAG
CTTGAGACTGAACAAGCTGTAAGGCCAAAATATCTGGCAGGAAAGGAAGTTACTGGGAAC
ATGCGTGCCATTCTTATCGACTGGCTTGTGCAAGTCCAGATTAAGTTTAGGCTGCTTCAG
GAGACCATGTACATGACTGTTGCCATCATCGATCGCTTCCTTCAGGATCATCCAGTTCCA
AAGAAGCAGCTGCAACTTGTTGGTGTAACGGCCATGTTCATCGCCTCAAAATATGAAGAA
ATGTACCCACCAGAGATTGCAGACTTTGCTTTTGTGACAGACCGTGCATACACCACCAGT
CAGATCCGGGAGATGGAGATGAAGGTCCTGAGAGTCCTCAACTTTGGTTTTGGAAGGCCT
CTACCACTACAGTTTCTCAGGAGGGCCTCCAAGATTGGAGATGTGACTGCAGAACATCAC
ACATTAGCGAAATATTTCCTGGAGCTCACCATGGTCGATTATGACATGGTCCACTACCCT
CCCTCTCAGATGGCCAGTGCTGCTTATGCCCTGACCCTGAAGGTCTTCAACTGTGGTGAC
TGGACCCCTACTCTTCAGCACTACATGGGCTACACTGAAGATGAGCTGGTTCCTGTGATG
CAGCATATTGCCAAAAATGTTGTGAGGGTCAACGAGGGCCTTTCTAAGCATCTGGCTGTG
AAGAATAAGTACTCCAGTCAGAAGCAGATGAGAATCGCTACAATTTCTCAGCTCAAGTCC
TCCTTGATCAAAGACCTGGCTAAGCAGATCTCATAGTTGGGGTTATGCTGAAGAGACTTA
ACGACTGTGTGCTGCCTTGTAAAAATTGTAACTTATGCATGTTTTACTGTTAAACTTTTT
ATTGCAATTTATTTTACGGATTTCTTCACTGCCATGGGTTGAGCAAAAGTAATCTTTTAA
TGATTCAAAGTGTTTATGCAAGTTTGTACATTTGAATTCAAATAAACTTTTTAAAGTTTT

Zebrafish *dazl*

GGTCGATTATGTGCATCTGTCAGGAGAGCATAAATATTCGTCCAATTAAGATTTTTTACC
CTAAATGCCCAGCTTGCCTAATGAAGAAACACGATAAGACCGTGTTCGGGATCTATCCGG
TTTCGGCCGCGTGTGGATTGAGCATTTAGAAAGTTAACGTTAGCGTTATTCGTTTAGCTT
TTCGACGGACTTTGGGGTTTGTTTTTGTAAAAAGCTTAGGTAGCAAAACGCGTGGCCTTG
TTTCTCATTCAAAATGGTTCAGGGGGTTCAGTTACCCGTGTGCCTGATATGTGGTTTGTA
TTCACAGGATATCCAGAAGCATCGTCAGGGTTTTCCGTCCTCCCTGAAGTTGTCTAACGG
TTACATTTTACCTGAGGGGAAAATGACGCCCAACACACTGTTCGTCGGCGGTATTGATAT
GAAGGTGGATGAGAACGAGATCAGGGAATTCTTTGCCAAGTATGGCTCAGTGAAAGAAGT
TAAAATCATCACTTATCGAGGAGGAATTTGCAAAGGATATGGTTTCGTTTATTTCAGTGA
GGATGTTGATATCCAGACTATCGTTGATCAGCCGATCAGTTTTAAAGGAAAAAACTCAA
ACTGGGACCTGCAATCATGAAAGAGCGAAGTTCTCGGTCAGTGTCATCTCCAATGATTGG
TCCATCACAGTGGGTAAACCCCACCCCATATATGTACTGCAGCTGCTGTCCTCCAGGCCT
GGCCCCACCATCACCCGTATTCAGTGGAGGAAATCAGTACATGCAGCCTTATTCTTACTC
CAGTCCTCCAGGAATTATGGTTCCACAGGTGCCAATGAACTATGCACAGACCACGTATGC
CTATCAGTATCCCCTGCCACAGTGGTGTGGAGAGCAAAGGACGAGGCTTGTCAATCAGAA
TTTTGTGGATTGTGGAGTGCAGACTTTGCTAACCCTTATGTAG

Mouse *Cyclin B1*

GGAACGGCTGTTAGTGTTTAGCTGTGGATAGCCAGAGGTTAGGGTGTCTTCTCGAATCGG

GGAACCTCTGATTTTGGAGGAGCCATGGCGCTCAGGGTCACTAGGAACACGAAAATTAAC
GCAGAAAATAAGGCCAAGGTCAGTATGGCAGGCGCCAAGCGTGTGCCTGTGACAGTTACT
GCTGCTTCCAAGCCCGGGCTGAGACCGAGAACTGCTCTTGGAGACATTGGTAATAAAGTC
AGCGAAGAGCTACAGGCAAGAGTGCCTCTGAAAAGGGAAGCAAAAACGCTAGGTACTGGA
AAAGGTACTGTTAAAGCCCTACCAAAACCTGTAGAGAAGGTGCCTGTGTGTGAACCAGAG
GTGGAACTTGCTGAGCCTGAGCCTGAACCTGAACTTGAACATGTTAGAGAAGAGAAGCTT
TCTCCTGAACCTATTTTGGTTGATAATCCCTCTCCAAGCCCGATGGAAACATCTGGATGT
GCGCCTGCAGAAGAGTATCTGTGTCAGGCTTTCTCTGATGTAATCCTTGCAGTGAGTGAC
GTAGACGCAGATGATGGGGCTGACCCAAACCTCTGTAGTGAATATGTGAAAGATATCTAT
GCTTATCTCCGACAACTGGAGGAAGAGCAGTCAGTTAGACCAAAATACCTACAGGGTCGT
GAAGTGACTGGAAACATGAGAGCTATCCTCATTGACTGGCTAATACAGGTTCAGATGAAA
TTTAGGCTGCTTCAGGAGACCATGTACATGACTGTGTCCATTATTGATCGGTTCATGCAG
AACAGTTGTGTGCCCAAGAAGATGCTACAGCTGGTCGGTGTAACGGCCATGTTTATTGCA
AGCAAATATGAGGAGATGTACCCTCCAGAAATAGGTGACTTCGCCTTTGTGACTAACAAC
ACGTACACTAAGCACCAGATCAGACAGATGGAGATGAAGATTCTCAGAGTTCTGAACTTC
AGCCTGGGTCGCCCTCTGCCTCTGCACTTCCTCCGTAGAGCATCTAAAGTCGGAGAGGTT
GACGTCGAGCAGCACACTTTGGCCAAATACCTCATGGAGCTCTCCATGCTGGACTACGAC
ATGGTGCATTTTGCTCCTTCTCAAATTGCAGCTGGGGCTTTCTGCTTAGCGCTGAAAATT
CTTGACAACGGTGAATGGACACCAACTCTGCAGCACTACCTATCCTACAGTGAAGACTCC
CTGCTTCCTGTTATGCAGCACCTGGCTAAGAATGTAGTCATGGTGAACTGTGGCCTCACA
AAGCACATGACTGTCAAGAACAAGTATGCAGCATCTAAGCATGCTAAGATTAGCACGCTG
GCACAGCTGAACTGTACACTAGTTCAGAATTTGTCTAAGGCCGTGACAAAGGCATAACTC
CAATAGACTGCTACATCTGCAGATGCAGTTGGCACCATGTGCCGCCTGTACATAGGATAC
CTACCGTGTTTACTTGCTCTTCAATAAAGGTTGTGACTTCTCATTTTACATAGCTTAACT
CATTTGAATGTTGTTGCTTCTGAGTTTAGGCTAACGGAAGTTGTCGAATTTAGGAGTATA
TTAAAAACTGCATCTAGTTTTAACAGTGGATCCAACTAATGTATATATCTGTAGCCTATA
TGTCTATATACATCCTTCACTGTGTGTCCTTATATCATCATGTCTTCTGCCTCACTCTAG
TTTAAACTCTAAATCTACCAGCTAGTCCTTTGTTCCATTTTCCAGTGGTTGCCACCTTTA
ACCACTGTCTCTTGGTTTGTCAACTTTCAGATCTGAAACCAAGTATCTTTTTTTATGTAA
TTATTTATTTGTTCTTAATTGGAAAATAGGATGTTCAAAATTAAAGGTGTGTTTTAAAAA
GAATTTGCCCCCAAGTCTCACTATCAACAGATAAGGGTGTATTCTTGTATATCCTGTATA
GATATAATCATGCATATACTCCCAAGGAGATATTTTTATATGGGTTCATTTTATCAACAG
TATTCCTATCAGCATTCCTTTCAATGCCTATATTGCATTTCCTAGTGTGAACAAACTGTG
TGTAACATAGTCATTCCCTCGGTGGGATTCAAGTGCATTCTCTCAGTGCCCTCCACAGTG
TTCTTAAATGATGTTTAATGTCTTGCTTGGCTTCATTCATAGTAGCTCTTCCAGGGGTGT
GCTTTGAATTCTGACAGCCAGATGGGTGTGGCTGCCACCATACCAAGGCGCCACTCCTGT
CTTGTAATGCCACCTGGAAAAGAATCCTGTCTCATTTGCTGTTTTAATTTATACATCTGA
TATCAAGTTGAATAAAATTTATTGGTGGAAAGCTTT

Mouse *Dazl*

AGTGACTCGGCGACCCTCAGCACGCTCGCTTCGGGACTCCGCTGTCTCCGTGGCCGTGGG
CTCTCTTTCCACCACCGCCTCGGCTTTTTTTTGCCCGTTGGCCGGCTAGGCAGCCACCTC
ACGTAGCTGCCCCGTCGCGTGGCTTCTGCTCCACCTTCGAGGTTTTACCACCCGAACTCT
GGCCGCCATCATGTCTGCCACAACTTCTGAGGCTCCAAATTCAGCTGTCTCCAGGGAGGC
CAGCACTCAGTCTTCATCAGCAACCACAAGTCAAGGATATGTTTTGCCAGAAGGCAAAAT
CATGCCAAACACCGTTTTTGTTGGAGGAATTGATGTTAGGATGGATGAAACCGAAATCAG
GAGTTTCTTTGCCAGATATGGCTCAGTAAAAGAAGTGAAGATAATCACTGATCGAACTGG
TGTGTCGAAGGGCTATGGATTTGTCTCATTTTATAATGACGTGGATGTGCAGAAGATAGT
AGAATCACAGATAAATTTCCATGGTAAAAAGCTGAAACTGGGCCCTGCAATCAGGAAACA

AAATTTATGTACTTATCATGTGCAGCCACGTCCTTTGATTTTTAATCCTCCTCCTCCACC
ACAGTTCCAGAGTGTTTGGAGTAGTCCAAATGCTGAGACTTACATGCAGCCTCCAACCAT
GATGAATCCTATCACTCAGTATGTTCAGGCATATCCTCCTTATCCAAGTTCACCAGTTCA
GGTCATCACTGGATATCAGCTGCCTGTTTATAACTACCAGATGCCACCGCAGTGGCCTGC
TGGAGAGCAGAGGAGTTATGTTATACCTCCGGCTTATACAACTGTTAACTACCACTGCAG
TGAAGTTGATCCAGGAGCTGATATTTTGCCCAATGAATGTTCAGTTCATGATGCTGCTCC
AGCTTCTGGAAATGGCCCGCAAAAGAAGTCTGTGGACCGAAGCATACAGACAGTGGTCTC
TTGTCTGTTTAACCCTGAGAACAGACTGAGAAACTCTCTTGTTACTCAAGATGACTACTT
CAAGGATAAAAGAGTACATCACTTCAGAAGAAGTCGGGCAGTGCTTAAATCTGATCATCT
CTGC

Mouse *lncRNA-HSVIII*

TTCATCTTCCCTCACTAGTCATCATTTGCATATTCTCTTTTTTAAAGTGTTCTTTGTCCA
GATAATTTTTATTGCTGATTTTTTAAAGCTATGCTTCATATATTCTGATAAGAGTATTAC
ATCTAATCTTAGTTTGCTTTTAACTGGAGGTTGTGAAGAATTTTCTGTAATTCTTTTTCC
CCTAAGAAAAAGTGTTTCTAGTTTTAGCCTTGACACTTATGTCTGTGATTAACAAGTTAA
TTTTTGTTTGTGGCACATGGCAGAGATCAGAGTTTGTATTTCGACGCCTTTAATGCTCAT
TAAATGGTTGCAATCTGATTTGTTGACATGCATTTGTTTCAACATCAGTTTGAACCACTA
ATTGACAGTATATTTCTGCACCTGTTCCTTGACTTGATATCTGTAGACATAACTTTTTTT
CAGCCTACTTTTAATAAGGGTTCAACCTCTCCTCTAGCTCACCACCCAGCAGAGGTAGTG
GAAGAGAACAGTTATTAGGCTATGGGGGAAGTGGATCTGTTCAGCAAGAGTTCTTTGGGG
GCGAGCTCAGTCTTCCTTGGCAGCAGCCCAGTCCCATGGCAAACACCAAATGTGACTCAG
CAGCTGCAGACCAGTCCTCTAGGCAGGCAGACACCAGGCATGAACCAGCAACTGTAATTC
AATCCTGAAGAAACCAAGGCTCACCAACTGGCCTGAGGAGAGGCCGCAGAAGCAGCAAGC
TGCCTAAGGAACCTCATGAGCAGTTCTTTGGCCAGTTTCTCTCAATGGCATCACTAGTTG
AGATCAACAATGCTATGTAGGGCAAACCATACACCAACAAATACACGCATGTCACTGTAT
AGCGAGGTGGAGAAAATCAGACCAAGGCTCAGTGCTGTCTCCCACGCTCTGTGGGGTCAT
AGTTATACTCCTTCATGGCAGGTCCTTTCACGTGCTTGCTATATCAAAACATCCTTTCAC
CTGTGTGGCCTCAGGAAAACATTCTTTTCACATGTCTGTTTTAGCAAGACATCCCTTCAC
CTGTGTGTCCCAGCAAAACATCATTTGATGTAACTTAACTTTCCAAAGAAACTAGAAGTT
TCCACTTCACATATCTATCTACATATTCTTAAGTACAATACAGCTCTATCCCAGATATTG
TGACTTGTGATCTTGTGATTCGGTAGTGTTGTACTTACAGGTTTATTCACATTTTTCAAG
CTGCAGTCTTCTTGGAACTTATATGTTGATTCTAAAATGTGTATGGAGGGCTTCAGTGAC
AGCTCAGTTTGTAAAGTGCTTGCTGCATCTGCAACCATGATGACTTGAGTTCAACCCCCA
GCCCCACATAAAAAGCCAGGCATGGTGGTAGGTACCTATGATCCCAAGGGCTGGAGAGGC
AGAAGCCAGTAGACCTCTGGGACTGCTTAGGCAGCCAGCTCAGCCTAACAGACAAATCCA
GACCCAGTGAGAGGCTCTGTCTCAAAAACGCAAAGGGAACAGCTTTGGAGGTGCAATACC
CAAGGGTGACGTGTGGCCTCCGTGCTCAACACTTGAGCACATGTACCTGCACATACCTGA
ATACACACTTGTATGTAAACACACAGATAAAATGGCTGGAGTGATGGCTTAGTGGTTAAG
AGTGCTTGCTACTTTCCTAGAGGGCCTGAGTTTGGTTCCCAGCACCCGTGTCCAAGCTGC
TCAGAACCATCTGCAACTCTAGTTCCAGAGTATCTGGCCTCTAAGGGCACCTGCACATGT
GTTTGCATATACACTCATAAATAAAAATAAAATAAATCTTTAAAAATGTCGCGCCCGCTC
TCGGCCAGCAAGAACGACGCGACCACCAGTCCTTCTAACAGCAGTTTATTCAGTCCTGAT
TCTTCTTGTTTATATCTCCCCTGAACCCTGGGCCTCTCACTCCTTTTATACTCTCTCTCA
TCCACGCACCGCAGGCCACGCCCCCTCGCCAGTCACGAGGCTTCAGCTAATCAGGGCAGC
AGGGGCAAATCTCCACCAAATTGGATTCACCTGTATCCTGGTACACCTGCGCAGCACTCA
AGATGTTTGTGTCTTATATGAGGAAGTCAGGTGCAAGTCATATGACTTAGCTGCAGTCCC
TGGCGCCTTTGGGACTGCCGCCACACCCGCTCCCCACATAAAAATGTATATTGAAAGATG
AAAAGACTCAGAATGACTAATACCAACAATATAGGTTTCAAGACTTTAAAATGATATGCC

AGTCAATACAGGGATGTGTTGTCATTGGGGCAGAAATATAGATCAGTAGAACAGAACAAG
GAATCTGGAAATAGGTCCACATCTATTCAGCCAGATGATCTTGATGAGAGTACTAATGTA
ACCCAATGGGGGAAAAGATAGTATTTTTACTTAGTAAATGATGCTGAGGGTTTAGGAATG
CACTTCAGTATAGAGGAGCTCTTATCTAGTGTGCATGAGGGCCTTAGTTTAATACCTAGC
ACCACAAAAACAAAGGGTGTTAGAACAGTGGCCTTTGTGCCTGGTGTTGGTTGCGCACAC
CTTTAATCCCAGCACTTGGGAAGCAGAGGCAGGTAGATCTCTGTAAGTTCGAAGCCAGCC
TGGTCTACACAGAGAGAGAGAGAGTTCCAGAACAGCCAGGGCTACACAGAGAAAAGAAAA
GAAAAGAAAAGAAAAGAAAAGAAAA

Open reading frame (ORF) sequences are highlighted with gray boxes.

Supplemental Figures Introduction

Supplemental figures show results of experiments independent from those shown in Figs. 2-6 and experiments using the control sense probes. Figure S1 shows results of experiments independent from those shown in Fig. 2C and D. The signals were detected in oocytes of sections hybridized with the antisense RNA probe but not in those hybridized with the sense RNA probe. Figure S2 shows results of experiments independent from those shown in Fig. 3 and experiments using the control sense probes. No signal was observed in the oocytes including the animal polar cytoplasm beneath the micropyle using the sense probes. Figure S3 shows results of experiments independent from those shown in Fig. 4 and experiments using the control sense probes. No signal was observed in the all stages of oocytes using the sense probes. Figure S4 shows results of experiments independent from those shown in Fig. 5 and experiments using the control sense probes. No signal was observed in the oocytes using the sensel probes. Figure S5 shows results of experiments independent from those shown in Fig. 6 and experiments using the control sense probes. Slight signals were observed in the sections hybridized with the sense probes, showing fluorescence background in this procedure. In contrast, bright dot-like signals were observed in the nucleus of spermatocytes and the cytoplasm of round sperms in sections hybridized with the antisense probes. Explanations of supplemental figures in details are similar to those of Figs. 2-6.

Supplemental Figure Legends

Figure S1. Single *in situ* hybridization of *Pou5f1/Oct4* mRNA in mouse ovaries. (A-D) Sections hybridized with the sense (A and C) or antisense (B and D) *Pou5f1/Oct4* RNA probe with amplification of signals using the TSA system (+TSA). Bars: 100 μm.

Figure S2. Double fluorescence *in situ* hybridization of *mos* and *cyclin B1* mRNAs in zebrafish ovaries. (A-C) A section hybridized with the sense probes for *mos* and *cyclin B1* mRNAs in a fully grown oocyte. (D-F) A section hybridized with the antisense probes for *mos* and *cyclin B1* mRNAs in a fully grown oocyte. Bars: 50 μm in A-C, 10 μm in D-F.

Figure S3. Double fluorescence *in situ* hybridization of *cyclin B1* and *dazl* mRNAs in zebrafish ovaries. (A-D) Sections hybridized with the sense probes for *cyclin B1* and

dazl mRNAs. Follicles consisting of stage Ia (A), stage Ib (B), stage II (C), and stage III (D) oocytes. (E-H) Sections hybridized with the antisense probes for *cyclin B1* and *dazl* mRNAs. Follicles consisting of stage Ia (E), stage Ib (F), stage II (G), and stage III (H) oocytes. Bars: 20 μm in A, B, E and F, 50 μm in C, D, G and H.

Figure S4. Double fluorescence *in situ* hybridization of *Cyclin B1* and *Dazl* mRNAs in mouse ovaries. (A) A section hybridized with the sense probes for *Cyclin B1* and *Dazl* mRNAs. (B and C) Sections hybridized with the antisense probes for *Cyclin B1* and *Dazl* mRNAs. Bars: 20 μm.

Figure S5. Fluorescence *in situ* hybridization of *lncRNA-HSVIII* in mouse testes. (A-B) Transverse sections of seminiferous tubules hybridized with the fluorescein-labeled sense (A) and antisense (B) probes. (C-F) Transverse sections of seminiferous tubules hybridized with the DIG-labeled sense (C and E) and antisense (D and F) probes. Bars: 50 μm.

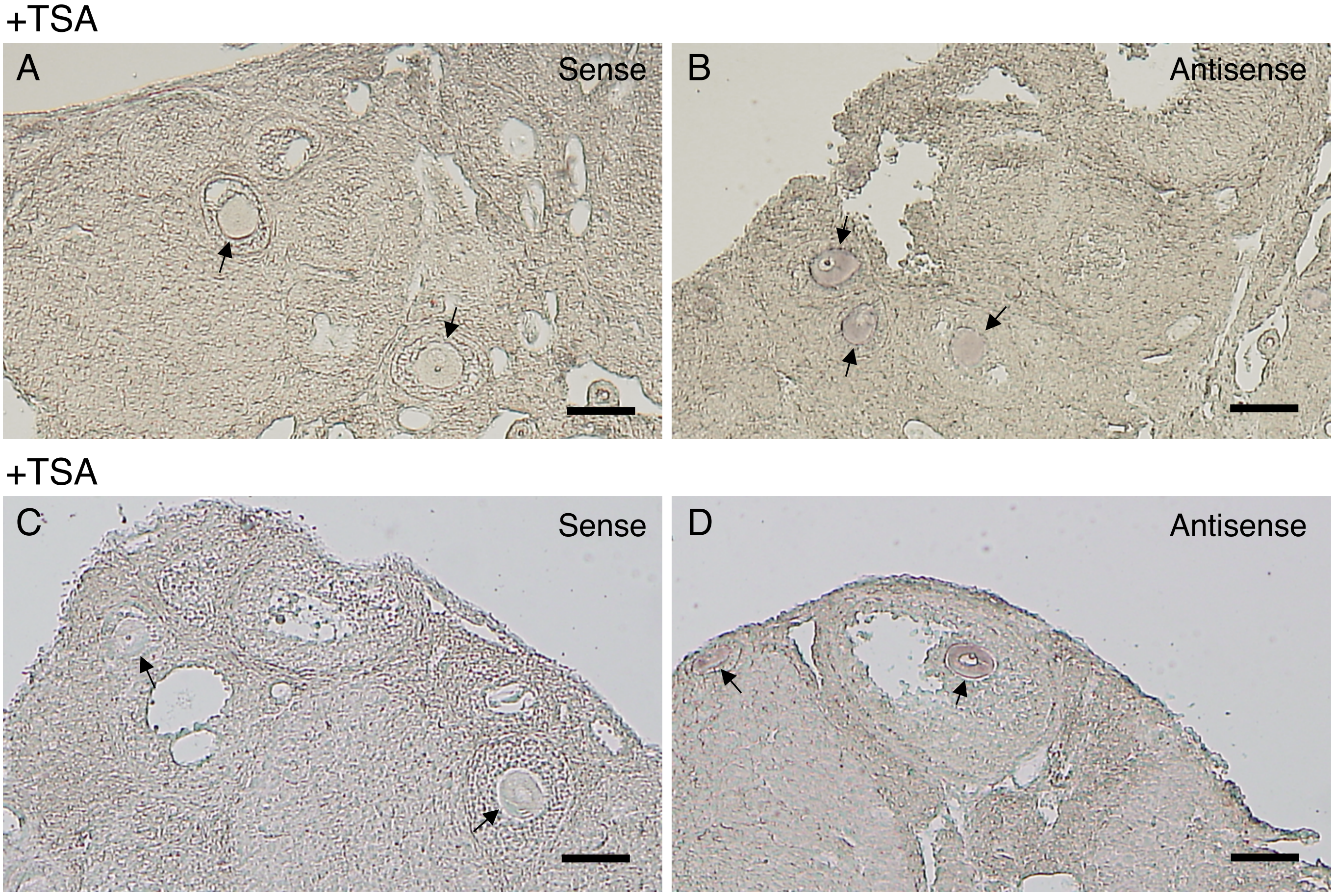

Figure S1

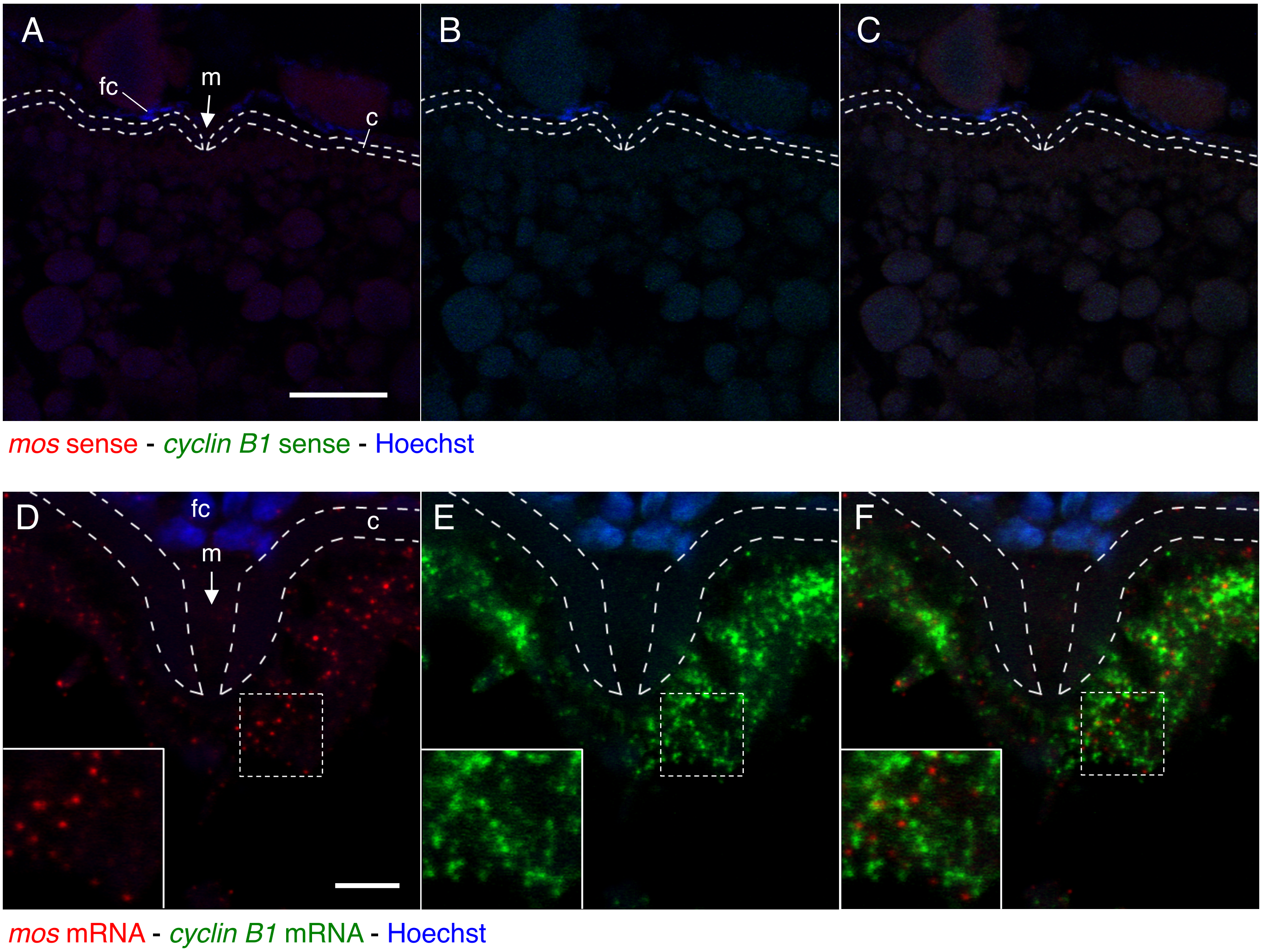
A
fc
m
c
B
C
mos sense - *cyclin B1* sense - Hoechst
D
fc
m
c
E
F
mos mRNA - *cyclin B1* mRNA - Hoechst

Figure S2

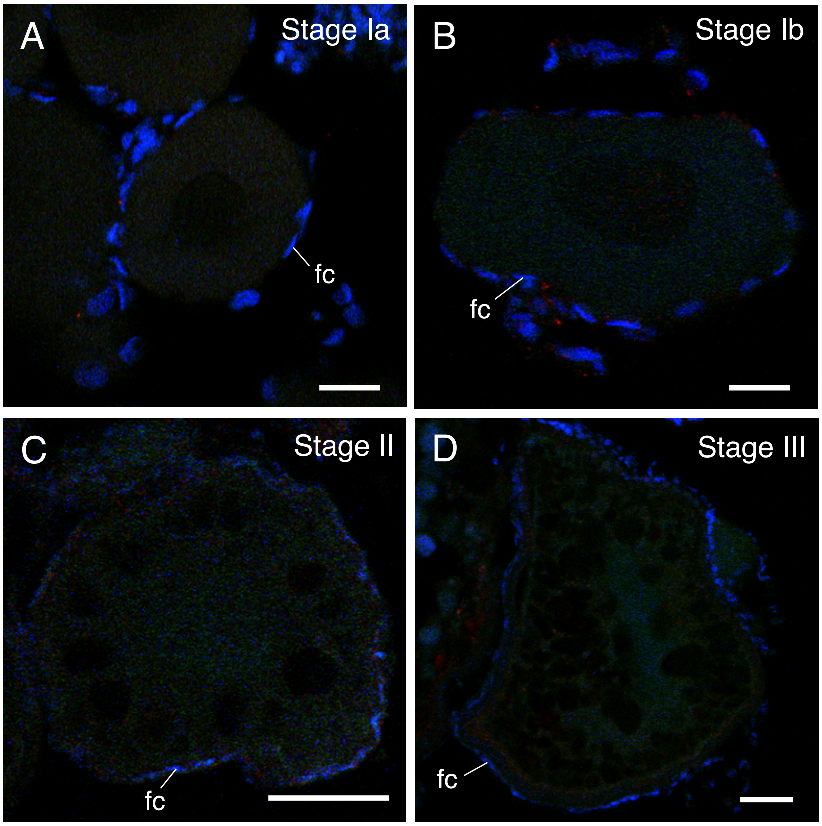

cyclin B1 sense - *dazl* sense - Hoechst

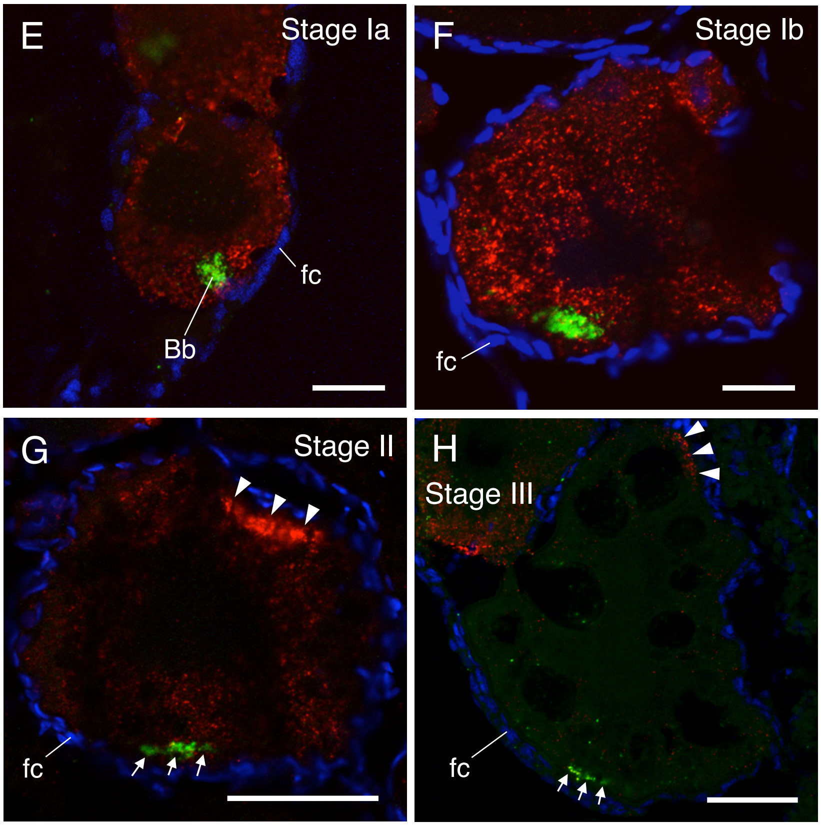

cyclin B1 mRNA - *dazl* mRNA - Hoechst

Figure S3

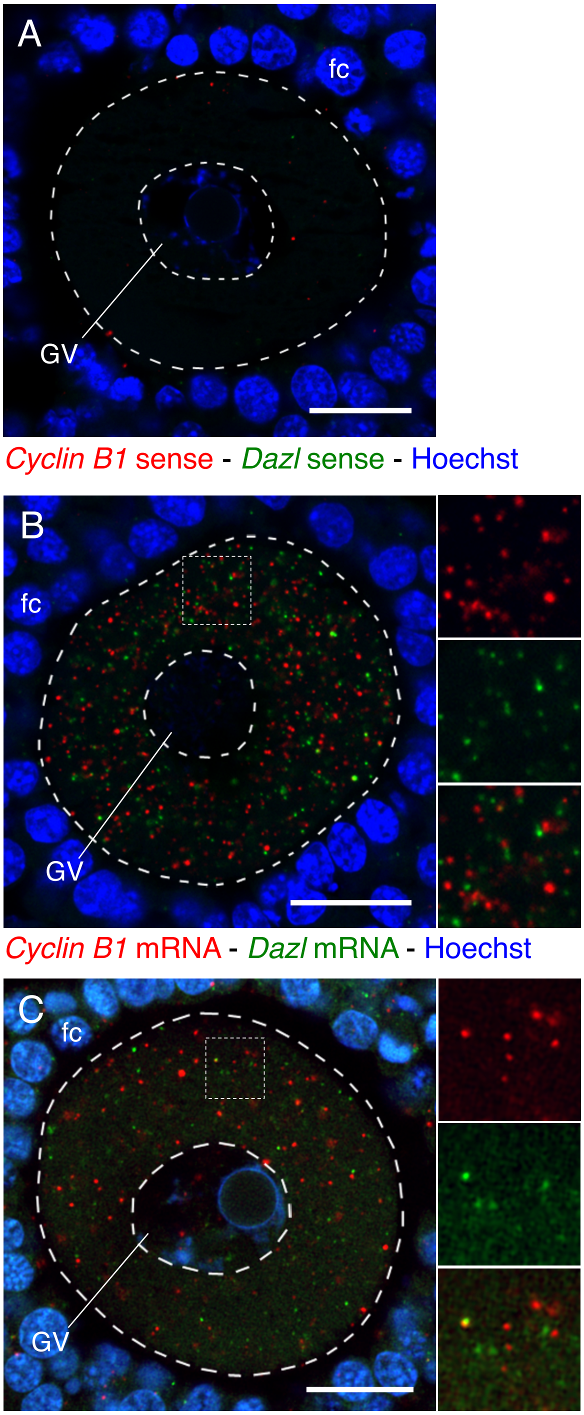

Cyclin B1 sense - *Dazl* sense - Hoechst

Cyclin B1 mRNA - *Dazl* mRNA - Hoechst

Cyclin B1 mRNA - *Dazl* mRNA - Hoechst

Figure S4

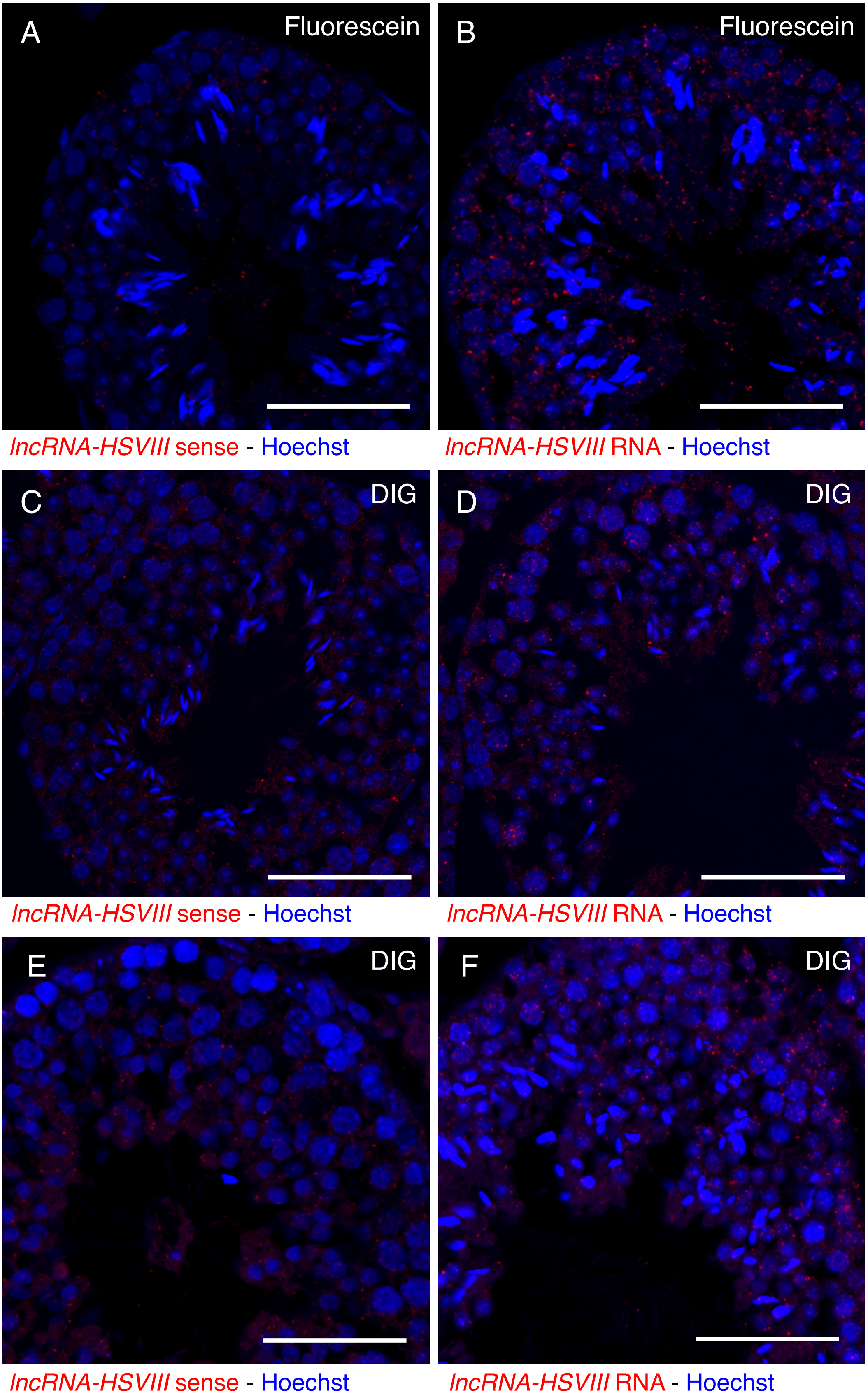
A
Fluorescein
B
Fluorescein
lncRNA-HSVIII sense - Hoechst
lncRNA-HSVIII RNA - Hoechst
C
DIG
D
DIG
lncRNA-HSVIII sense - Hoechst
lncRNA-HSVIII RNA - Hoechst
E
DIG
F
DIG
lncRNA-HSVIII sense - Hoechst
lncRNA-HSVIII RNA - Hoechst

Figure S5
